# Tasks of COVID-19 prevention and control management teams at primary health care facilities in mainland China: a nationwide online cross-sectional survey

**DOI:** 10.1186/s12875-022-01703-0

**Published:** 2022-05-06

**Authors:** Yun-yun Yan, Jian-li Ge, Teng-yang Fan, Hai-tang Wang, Yan-feng Gu, Xue Xiao, Zhao-hui Du, Xiao-ming Sun

**Affiliations:** 1 Department of General Practice, Zhongshan Hospital, Fudan University, 180 Fenglin Road, Xuhui District, Shanghai, China; 2 Department of General Practice, Shanghai East Hospital, Tongji University School of Medicine, Shanghai, 200120 China; 3 Department of General Practice, Affiliated Hospital of Zunyi Medical University, 149 Dalian Road, Huichuan District, Zunyi, Guizhou Province China; 4 Pudong New District Shanggang Community Healthcare Center, 360 Changli Road, Pudong New District, Shanghai, China

**Keywords:** Team members, Primary healthcare facilities, COVID-19, Prevention and control, Tasks, Mainland China

## Abstract

**Background:**

This research aimed to investigate the tasks performed by Coronavirus Disease 2019(COVID-19) prevention and control management teams at primary healthcare (PHC) facilities during COVID-19 pandemic across the mainland China.

**Methods:**

An online survey was performed and COVID-19 prevention and control management teams at PHC facilities were invited to participate in this research. The top 7 most important tasks in the three different periods of COVID-19 containment were selected and ranked. Participations of tasks were surveyed.

**Results:**

A total of 998 valid responses (an effective rate of 99.11%) were collected. The respondents were divided into Group A (≤5 respondents within each PHC facility, n_1_ = 718) and Group B (> 5 respondents within each PHC facility, n_2_ = 280). The consensus was selected from top 7 most important tasks including screening at travel centers/intervals and screening at entry centers, at-home/centralized quarantine management, transferring, pre-examination/triage and fever sentinel surveillance clinic/fever clinic. Pre-examination/triage and fever sentinel surveillance clinic/fever clinic works became more significant in the regular prevention and control period. Adjusted analysis found that team members of Group A with a college, undergraduate college and graduate school educational background were less involved in pre-examination/triage works (aOR: 0.28; 95%CI: 0.09-0.86, *P* = 0.026; aOR: 0.30; 95%CI: 0.10-0.90, *P* = 0.031; aOR: 0.21; 95%CI: 0.05-0.82, *P* = 0.024). Those who were over the median age were twice more likely to be engaged in managing fever sentinel surveillance of clinic/fever clinic visitors (aOR: 2.18; 95%CI: 1.16-4.08, *P* = 0.015). Those being specialized in nursing and other specialties were less likely to participate in fever sentinel surveillance of clinic/fever clinic works (aOR: 0.44; 95%CI: 0.24-0.81, *P* = 0.009; aOR: 0.30; 95%CI: 0.16-0.58, *P <* 0.001). Those came from central and western China were less likely to participate in centralized quarantine management (aOR: 0.61; 95%CI: 0.38-0.98, *P* = 0.042; aOR: 0.64; 95%CI: 0.42-0.97, *P* = 0.037). Team members came from central and western China were twice less likely to participate in screening at travel centers/intervals (aOR: 1.75; 95%CI: 1.14-2.70, *P* = 0.011; aOR: 1.63; 95%CI: 1.07-2.48, *P* = 0.024).

**Conclusion:**

In mainland China, team members of COVID-19 prevention and control at PHC facilities are mainly responsible for screening, quarantine, transferring and monitoring during the COVID-19 pandemic. Pre-examination/triage and the fever sentinel surveillance clinic/fever clinic were gradually valued. Team members with lower educational background are competent in pre-examination/triage works, but more experienced general practitioners are more likely to be in charge of fever sentinel surveillance clinic/fever clinics work. The necessity of COVID-19 prevention and control management teams to participate in screening at travel centers/intervals is subjected to further discussions.

**Supplementary Information:**

The online version contains supplementary material available at 10.1186/s12875-022-01703-0.

## Background

In the Coronavirus Disease 2019(COVID-19) pandemic, primary health care (PHC) systems, the first line of defense, faced unprecedented challenges [1]. Primary care worldwide played an important role in the struggling for COVID-19 containment [2, 3]. Despite the limited capacity in dealing with severe COVID-19 infections, well-organized PHC systems reduced the spread of the severe acute respiratory syndrome coronavirus 2(SARS-CoV-2), treated individuals with fever or respiratory symptoms, and further decreased the morbidity and mortality of this highly contagious disease during its pandemic [4, 5].

In summary, there were two major aspects of healthcare work that primary care providers (PCPs) performed during COVID-19 pandemic globally. The first role PCPs played worldwide is to meet the citizens’ medical care needs as usual. Owing to the strong infectivity of SARS-CoV-2, some innovative diagnosis and treatment strategies were initiated. Telehealth and remote consultations were promoted to protect health care workers (HCWs) and reduce viral transmission by minimizing face-to-face visits [6]. Although normal diagnosis and treatment process were hindered due to the sever situation resulted from COVID-19 pandemic, tele-consultation allowed PCPs across the world to communicate at a distance with people who needed healthcare service [7]. Then daily clinical diagnosis and treatment provided to the public were still methodically delivered.

On the other hand, as the “gatekeepers” of healthcare, PCPs also engaged in dealing with COVID-19 related high-risk populations as expected. COVID-19 patients’ identification and triage were also key elements for PHC workers [8]. According to World Health Organization (WHO), PCPs were in charge of distinguishing mild, moderate and severe cases [9]. During the pandemic of COVID-19, consultations with PCPs were recommended firstly for febrile patients with/without any respiratory symptoms and individuals who were at the risk of getting infected with SARS-CoV-2 in Australia [10]. In Singapore, a total of 942 PHC centers participated in the early identification of COVID-19 patients, and 32% of the confirmed cases were identified at PHC centers [5]. In Atlanta, USA, 83.2% of discharged COVID-19 patients made appointments with a primary care provider [3]. In addition, the primary healthcare providers (PCPs) were also the potential teams in providing health care management at home for patients with mild COVID-19 symptoms, and managing patients while waiting for specialized hospital beds [4]. All the tasks mentioned above were COVID-19 related specific works fulfilled by PCPs.

Moreover, except for management of high-risk populations, community containment measures such as quarantine, border closures and travel restrictions were also the main topic in the COVID-19 pandemic control process [11, 12]. In some European countries, public health departments were mainly responsible for the community containment activities [13]. However, in China, PCPs are involved in not only high-risk population screening and management, but also the travel restrictions and quarantines both at-home and at centralized centers.

As the COVID-19 pandemic situation changed over time, the curve of COVID-19 pandemic in China flattened since early April, 2020. There were some differences in the tasks of PCPs compared with other countries. This survey aimed to investigate the tasks conducted by team members of COVID-19 prevention and control at PHC facilities and members associated with these tasks during the COVID-19 pandemic across the mainland China.

## Methods

A cross-sectional survey was performed to investigate the tasks of primary care during the COVID-19 pandemic in mainland China, and the online questionnaire was distributed among PCPs from different PHC facilities across the country.

### Questionnaire development

Firstly, comprehensive literature search in English and Chinese electronic database were conducted to learn the community prevention and control tasks against COVID-19 pandemic domestically and globally. Subsequently, we interviewed 5 PCPs from Shanghai and Zunyi, Guizhou province and designed the original version of questionnaire. Then, we conducted face-to-face pilot interview from November to December, 2020. At 16 PHC facilities located in urban, urban-rural and rural areas of three selected cities (Shanghai- medium risk city of eastern China, Wuhan, Hubei province- high risk city of central China and Zunyi, Guizhou province-low risk city of western China), 32 members of COVID-19 prevention and control management team at PHC facilities were interviewed following the topic presented in original version of questionnaire. According to the advice of interviewees, we revised the questionnaire and eventually adapted it into an online version.

The questionnaire was constructed and divided into 3 sections: 1) the basic demographics and professional specialties of the participants, including 9 questions; 2) selection and rank of the top 7 most important tasks from the total of 19 tasks for the three different periods of COVID-19 pandemic, referred to pre-outbreak period, outbreak period and regular prevention and control period. One supplementary answer was allowed or required if the task the respondents thought important was not included in the 19 tasks listed; 3) survey of the tasks participated by the respondents during the COVID-19 pandemic, including 9 questions (see Supplementary Materials).

The questionnaire was revised later according to the advice of interviewees, thus the final version should have a higher degree of validity than the original one. In addition, in the final version of the questionnaire, a supplementary answer was allowed by the respondents in case of any crucial items were missed in our questionnaire. However, the lack of new topic raised by the respondents further supported the content validity of the questionnaire**.**

### Sample size

The sample size of PHC facilities was determined according to the calculation below. Based on the pilot field survey *P* = 0.5, d and α was set at 0.05, thus the estimated minimum sample size was 385. To increase the validity of questionnaire, we enlarged the sample size by 20% and the final sample size reached 462.

$$n=\frac{Z_{\frac{\alpha }{2}}^2\bullet P\left(1-P\right)}{d^2}$$

### Participants

Following literature search, tele-interview and pilot field survey, we treated team members of COVID-19 prevention and control management at PHC facilities as target samples to meet program-specific requirements.

Purposive stratified sampling was used in participant selection, and all of the 31 provinces & regions & Xinjiang Production and Construction Corps (excluded Taiwan, Hongkong and Macau) in mainland China were included. Firstly, 5 city& districts were selected in each provinces & region, especially the city& district which was at high- or medium-risk during the COVID-19 pandemic. Secondly, in each city& district, 3 PHC institutions were chosen from its urban, urban-rural and rural areas. Sampling of PHC institutions was nonrandom and purposive. Next, team members of COVID-19 prevention and control management of each PHC facility were invited to complete the questionnaire. All of the participants were fully engaged in preventing and containing COVID-19 throughout the pandemic period. An online survey was carried out and the questionnaire was distributed by WeChat and website hyperlinks. The PHC facilities selected for the pilot interviews were avoided to participate in the online survey.

An electronic consent form was provided before the beginning of the online survey. Once the informed consent was obtained, the questionnaire was valid. The president of reginal community health association from each provinces & regions was invited to ensure that the questionnaire distribution covered the PHC institutions from typical areas. For example, the PHC facilities from high- or medium-risk areas (the risk areas were defined by the number of confirmed cases during the last 14 days) were focused on in purposive stratified sampling. During the survey, two well-trained researchers were assigned to monitor the submission in case of over- or under-respondence in each region.

The online survey was carried out from February 22, 2021 to March 2, 2021.

### Data analysis

All the data collected via online survey were extracted into an Excel spreadsheet using Microsoft Excel (Version 2019). Statistical analysis was performed using SPSS (IBM SPSS Version 22.0, SPSS Inc). The enumeration data were tested using *chi-square* test. A logistic regression model was employed to identify determinants of task participation. In the first step, associations between explanatory variables and task participation were analyzed. In the second step, all variables with *p* ≤ 0.25 in the first step were included in the adjusted analysis. The significance of crude odds ratio (OR) from univariate analyses and adjusted OR (aOR) in multivariate analyses were assessed. Size of a test was 0.05 (α = 0.05).

To assist in selecting the top 7 tasks important in the pre-outbreak period, outbreak period and regular prevention and control period of COVID-19 pandemic, we calculated a composite index for each task selected by the respondents according to the following formula:

Composite score = $$\frac{\left(\sum frequency\times weights\right)}{Total\ number\ of\ participants\ selected\ that\ item.}$$

The formula took into consideration of the respondents’ personal prioritization on each task. The importance degree was ranked according to the weights 7 to 1, with the score of 7 being the most important task and 1 being the seventh important task. For example, the composite score of screening at travel centers/intervals in pre-outbreak period of COVID-19 pandemic was 6.02 [= (348 × 7 + 19 × 6 + 13 × 5 + 25 × 4 + 23 × 3 + 23 × 2 + 23 × 1)/474, i.e., 348 respondents selected this task as the most important one and 23 respondents selected it as the seventh important task].

## Results

A total of 1007 participants from 421 PHC facilities submitted the questionnaire, of which 1001 agreed to participate in the survey and 998 completed the questionnaire (an effective rate of 99.11%).

Preliminary data analysis showed that 280 respondents from 13 PHC facilities submitted questionnaires and the least number of respondents was 6 of each facility. However, in the pilot field survey, we learned at most 5 team members of COVID-19 prevention and control management were hired at each PHC institution. Then we compared the descriptive characteristics between the 280 and the other 718 respondents, and significant differences between the two groups were revealed (Table 1). Therefore, the 280 respondents were analyzed individually as a subgroup names Group B to differentiate from the others named Group A. Logistic regression analysis was used for regression and correlation analyses between the two groups (Table S1).

**Table 1:** Descriptive characteristics of respondents

Characteristics	No. of Group A (%; *N =* 718)	No. of Group B (%; *N =* 280)	***P value***
**Age (yr) Median (IQR)**	40 (34-45)	33 (26-43)	< 0.001*
**Sex**			
Male	277 (38.58)	52 (18.57)	< 0.001*
Female	441 (61.42)	228 (81.43)	
**Education**			
Technical secondary school	30 (4.18)	29 (10.36)	< 0.001*
College	182 (25.35)	130 (46.43)	
Undergraduate college	473 (65.88)	110 (39.29)	
Graduate school	23 (3.20)	3 (1.07)	
Others	10 (1.39)	8 (2.86)	
**Profession**			
Clinical medicine	138 (19.22)	7 (2.5)	< 0.001*
General medicine	204 (28.41)	49 (17.5)	
Traditional Chinese medicine	60 (8.36)	33 (11.79)	
Nursing	192 (26.74)	113 (40.36)	
Others ^a^	124 (17.27)	78 (27.86)	
**Technical title**			
Junior	246 (34.26)	164 (58.57)	< 0.001*
Intermediate	253 (35.24)	61 (21.79)	
Associate senior	150 (20.89)	8 (2.86)	
Senior	25 (3.48)	0	
Others ^b^	44 (6.13)	47 (16.79)	
**Years of work experience Median (IQR)**	14 (8-23)	10 (4-23)	< 0.001*
**Regions**			
Eastern	263 (36.63)	50 (17.86)	< 0.001*
Central	196 (27.30)	46 (16.43)	
Western	259 (36.07)	184 (65.71)	
**Intro-city locations**			
Urban	343 (47.77)	163 (58.21)	< 0.001*
Urban-rural	187 (26.04)	88 (31.43)	
Rural	188 (26.18)	29 (10.36)	
**Risk areas**			
Low-risk	645 (89.83)	272 (97.14)	< 0.001*
Medium-risk	45 (6.27)	50 (5.01)	
High-risk	28 (3.90)	31 (3.11)	

^a^ including public health, preventive medicine, laboratory medicine, imaging, stomatology, paramedical and non-medical specialty

^b^ non-medical technical title or no title

*IQR* Interquartile range. *P < 0.001

### Consensus on the top 7 most important tasks

In China, the process of COVID-19 pandemic was divided into three periods. The pre-outbreak period referred to the period from the initial report of unexplained pneumonia cases to the strict travel restrictions in Wuhan, ranging from December 31,2019 to January 23,2020. The outbreak period ranged from January 23, 2020 to March 17, 2020 [14], whereas the regular prevention and control period began after March 17, 2020, when no newly infected patients were identified in Hubei Province.

Table 2 lists the top 7 most important tasks in different periods of COVID-19 containment, in rank order by composite score. All tasks selected by two groups were categorized into screening (2 items including screening at travel centers/intervals and screening at entry centers), quarantine (2 items including at-home/centralized quarantine management), transferring (1 item), management measures fulfilled within PHC facilities (3 items including pre-examination/triage, prevention and control of nosocomial infection and fever sentinel surveillance clinic/fever clinic), and routine clinical work (1 item). In the table, control of nosocomial infection was selected by Group A and routine clinical work was selected by Group B during the pre-outbreak period respectively.

**Table 2:** The top 7 most important tasks of PHC facilities in different periods of COVID-19 containment, China

Rank	Tasks ranking by Group A	Composite score	Tasks ranking by Group B	Composite score
**Pre-outbreak period**				
1	Screening at travel centers/intervals	5.84	Screening at travel centers/intervals	6.38
2	Screening at entry centers	5.48	Screening at entry centers	5.87
3	At-home quarantine management	5.110	At-home quarantine management	5.23
4	Pre-examination/triage	5.108	Pre-examination/triage	4.69
5	Centralized quarantine management	4.69	Centralized quarantine management	4.51
6^a^	Prevention and control of nosocomial infection ^b^	4.30	Transferring	4.22
7^a^	Transferring	4.21	Routine clinical work ^b^	3.98
**Outbreak period**				
1	Screening at travel centers/intervals	5.84	Screening at travel centers/intervals	6.37
2	Screening at entry centers	5.52	Screening at entry centers	5.74
3	At-home quarantine management	5.30	At-home quarantine management	5.46
4	Pre-examination/triage	4.95	Pre-examination/triage	4.81
5	Centralized quarantine management	4.72	Centralized quarantine management	4.63
6	Transferring	4.14	Transferring	4.42
7	Fever sentinel surveillance clinic/fever clinic ^b^	4.09	Fever sentinel surveillance clinic/fever clinic ^b^	3.97
**Regular prevention and control period**				
1	Screening at travel centers/intervals	5.99	Screening at travel centers/intervals	6.41
2^a^	Pre-examination/triage ^b^	5.73	Screening at entry centers	5.48
3^a^	Screening at entry centers	5.67	At-home quarantine management	5.42
4^a^	At-home quarantine management	5.32	Pre-examination/triage	5.34
5^a^	Fever sentinel surveillance clinic/fever clinic ^b^	4.81	Centralized quarantine management	4.70
6^a^	Centralized quarantine management	4.72	Transferring	4.57
7^a^	Transferring	4.61	Fever sentinel surveillance clinic/fever clinic	4.44

^a^ Difference between Groups

^b^ Difference between periods

In outbreak and regular prevention and control period, screening of high-risk individuals among PHC facility visits via pre-examination/triage, and the febrile patients’ management at the fever sentinel surveillance clinic/fever clinic became more significant for PHC facilities in the fighting against COVID-19 for both groups of respondents (Table 2).

### Task participation

Table S2 showed the comparison of task participation between the two groups. The adjusted analysis found that all the other 7 tasks were more likely to be participated in by Group A except for screening at entry centers (Table S3). The determinants of every task participation within the two groups were analyzed respectively.

### Associated determinants of task participation in group a

The adjusted analysis found that among members of Group A, those who were from high-risk areas were tripled the odds of participating in patient treatment compared to those who were from low-risk areas (aOR: 3.28; 95%CI: 1.21-8.87, *P* = 0.019) (Table 3).

**Table 3:** Task participation among team members of Group A during COVID-19 containment in mainland China

Characteristics	Task participation		Unadjusted		Adjusted	
	No [n(%)]	Yes [n(%)]	OR (95%CI)	***P value***	OR (95%CI)	***P value***
***Patient treatment***						
**Risk**						
Low-risk	552 (85.58)	49 (7.60)	1	–		
Medium-risk	39 (86.76)	3 (6.67)	0.87 (0.26-2.90)	0.819	0.65 (0.19-2.28)	0.500
High-risk	26 (92.86)	8 (28.57)	4.87 (2.04-11.61)	< 0.001*	3.28 (1.21-8.87)	0.019*
***Pre-examination/triage***						
**Educational level**						
Technical secondary school	4 (13.33)	26 (86.67)	1	–		
College	59 (32.42)	123 (67.58)	0.32 (0.11-0.96)	0.042*	0.28 (0.09-0.86)	0.026*
Undergraduate college	165 (34.88)	308 (65.12)	0.29 (0.10-0.84)	0.022*	0.30 (0.10-0.90)	0.031*
Graduate school	11 (47.83)	12 (52.17)	0.17 (0.04-0.64)	0.009*	0.21 (0.05-0.82)	0.024*
Others	3 (30)	7 (70)	0.36 (0.06-1.99)	0.241	0.37 (0.06-2.16)	0.272
**Intro-city location**						
Urban	110 (32.07)	233 (67.93)	1	–	1	
Urban-rural	71 (37.97)	116 (62.03)	0.77 (0.53-1.12)	0.172	0.62 (0.41-0.96)	0.032*
Rural	61 (32.45)	127 (67.55)	0.98 (0.67-1.44)	0.929	0.85 (0.55-1.32)	0.472
***Fever sentinel surveillance clinic/fever clinic***^***a***^						
**Age**						
At or below the median age	163 (57.39)	121 (42.61)	1	–	1	–
Above the median age	91 (37.76)	150 (62.24)	2.22 (1.56-3.15)	< 0.001*	2.18 (1.16-4.08)	0.015*
**Specialty**						
General medicine	32 (31.37)	70 (68.63)	1	–	1	–
Clinical medicine	61 (41.5)	86 (58.5)	0.64 (0.38-1.1)	0.105	0.71 (0.4-1.26)	0.244
Traditional Chinese medicine	18 (40.91)	26 (59.09)	0.66 (0.32-1.37)	0.267	0.68 (0.32-1.46)	0.322
Nursing	85 (59.86)	57 (40.14)	0.31 (0.18-0.52)	< 0.001*	0.44 (0.24-0.81)	0.009*
Others	58 (64.44)	32 (35.56)	0.25 (0.14-0.46)	< 0.001*	0.30 (0.16-0.58)	< 0.001*
***At-home quarantine management***						
**Specialty**						
General medicine	44 (31.88)	94 (68.12)	1	–	1	–
Clinical medicine	87 (42.65)	117 (57.35)	0.63 (0.40-0.99)	0.045*	0.71 (0.44-1.14)	0.156
Traditional Chinese medicine	31 (51.67)	29 (48.33)	0.44 (0.24-0.81)	0.009*	0.46 (0.24-0.86)	0.015*
Nursing	107 (55.73)	85 (44.27)	0.37 (0.24-0.59)	< 0.001*	0.44 (0.26-0.73)	0.002*
Others	59 (47.58)	65 (52.42)	0.52 (0.31-0.85)	0.010*	0.67 (0.39-1.15)	0.147
**Intro-city location**						
Urban	144 (41.98)	199 (58.02)	1	–	1	–
Urban-rural	96 (51.34)	91 (48.66)	0.69 (0.48-0.98)	0.039*	0.66 (0.45-0.95)	0.026*
Rural	88 (46.81)	100 (53.19)	0.82 (0.57-1.18)	0.284	0.70 (0.47-1.03)	0.073
***Centralized quarantine management***						
**Specialty**						
General medicine	73 (52.9)	65 (47.1)	1	–	1	–
Clinical medicine	137 (67.16)	67 (32.84)	0.55 (0.35-0.86)	0.008*	0.73 (0.46-1.18)	0.202
Traditional Chinese medicine	39 (65)	21 (35)	0.60 (0.32-1.13)	0.116	0.62 (0.32-1.19)	0.152
Nursing	140 (72.92)	52 (27.08)	0.42 (0.26-0.66)	< 0.001*	0.52 (0.30-0.89)	0.017*
Others	76 (61.29)	48 (38.71)	0.71 (0.43-1.16)	0.171	0.90 (0.52-1.55)	0.703
**Technical title**						
Junior	182 (73.98)	64 (26.02)	1	–	1	–
Intermediate	153 (60.47)	100 (39.53)	1.86 (1.27-2.72)	0.001*	1.86 (1.20-2.87)	0.005*
Associate senior	86 (57.33)	64 (42.67)	2.12 (1.38-3.26)	0.001*	1.96 (1.13-3.38)	0.016*
Senior	14 (56)	11 (44)	2.23 (0.97-5.17)	0.061	1.85 (0.70-4.88)	0.214
Others	30 (68.18)	14 (31.82)	1.33 (0.66-2.66)	0.425	1.30 (0.60-2.81)	0.500
**Region**						
Eastern	147 (55.89)	116 (44.11)	1	–	1	–
Central	136 (69.39)	60 (30.61)	0.56 (0.38-0.83)	0.003*	0.61 (0.38-0.98)	0.042*
Western	182 (70.27)	77 (29.73)	0.54 (0.37-0.77)	0.001*	0.64 (0.42-0.97)	0.037*
***Screening at travel centers/intervals***						
**Region**						
Eastern	191 (72.62)	72 (27.38)	1	–	1	–
Central	110 (56.12)	86 (43.88)	2.07 (1.4-3.07)	< 0.001*	1.75 (1.14-2.7)	0.011*
Western	173 (66.8)	86 (33.2)	1.32 (0.91-1.92)	0.148	1.63 (1.07-2.48)	0.024*
**Intro-city location**						
Urban	232 (67.64)	111 (32.36)	1	–	1	–
Urban-rural	139 (74.33)	48 (25.67)	0.72 (0.48-1.08)	0.109	0.57 (0.37-0.9)	0.016*
Rural	103 (54.79)	85 (45.21)	1.72 (1.2-2.49)	0.003*	1.3 (0.86-1.96)	0.211
***Screening at entry centers***	–	–	–	–	–	–
***Transferring***						
**Risk**						
Low-risk	516 (80)	129 (20)	1	–	1	–
Medium-risk	29 (64.44)	16 (35.56)	2.21 (1.16-4.19)	0.015*	2.45 (1.22-4.91)	0.011*
High-risk	17 (60.71)	11 (39.29)	2.59 (1.18-5.66)	0.017*	3.08 (1.28-7.42)	0.012*

^a^ Data of fever sentinel surveillance clinic/fever clinic not set were not involved in

Unadjusted and adjusted logistic regression analyses were used and variables in univariate analyses including: age, sex, educational levels, specialty, technical titles, years of work experience, economic area locations and intro-city locations of their PHC facilities, and the highest grade of risk levels the area of PHC facilities ever reached

Note: Only listed the results which were statistically significant in adjusted analysis. Table S4 showed all of the results which were statistically significant in un-adjusted analysis

*P < 0.05. *CI* Confidence interval

Members of Group A with a college, undergraduate college and graduate school educational background were less involved in pre-examination/triage works compared to those with a technical secondary school educational background (aOR: 0.28; 95%CI: 0.09-0.86, *P* = 0.026; aOR: 0.30; 95%CI: 0.10-0.90, *P* = 0.031; aOR: 0.21; 95%CI: 0.05-0.82, *P* = 0.024) (Table 3).

Those who were above the median age were twice more likely to engage in managing fever sentinel surveillance clinic/fever clinic visitors compared to those who were at or below the median age (aOR: 2.18; 95%CI: 1.16-4.08, *P* = 0.015); those being specialized in nursing and other specialties were less likely to participate in fever sentinel surveillance clinic/fever clinic works compared to those being specialized in general medicine (aOR: 0.44; 95%CI: 0.24-0.81, *P* = 0.009; aOR: 0.30; 95%CI: 0.16-0.58, *P <* 0.001) (Table 3).

Members of Group A being specialized in traditional Chinese medicine and nursing were less likely to participate in at-home quarantine management compared to those being specialized in general medicine (aOR: 0.46; 95%CI: 0.24-0.86, *P =* 0.015; aOR: 0.44; 95%CI: 0.26-0.73, *P =* 0.002) (Table 3).

Members of Group A being specialized in nursing were less likely to be responsible for centralized quarantine management compared to those being specialized in general medicine (aOR: 0.52; 95%CI: 0.30-0.89, *P =* 0.017); those came from central and western China were less likely to participate in centralized quarantine management compared to those came from eastern China (aOR: 0.61; 95%CI: 0.38-0.98, *P =* 0.042; aOR: 0.64; 95%CI: 0.42-0.97, *P =* 0.037) (Table 3).

Members of Group A came from central and western China were twice less likely to participate in screening at travel centers/intervals compared to those came from eastern China (aOR: 1.75; 95%CI: 1.14-2.70, *P =* 0.011; aOR: 1.63; 95%CI: 1.07-2.48, *P =* 0.024) (Table 3).

Those who were from medium- and high-risk areas were more likely to be involved in transferring compared to those who were from low-risk areas (aOR: 2.45, 95%CI: 1.22-4.91, *P =* 0.011; aOR: 3.08, 95%CI: 1.28-7.42, *P =* 0.012) (Table 3).

### Associated determinants of task participation in group B

Those being specialized in general medicine, traditional Chinese medicine and nursing were more likely to be responsible for pre-examination/triage works compared to those being specialized in other specialties (aOR: 2.54; 95%CI: 1.05-6.13, *P =* 0.038; aOR: 4.15; 95%CI: 1.50-11.50, *P =* 0.006; aOR: 3.63; 95%CI: 1.71-7.69, *P =* 0.001); those who were from central and western China were more likely to participate in pre-examination/triage works compared to those who were from eastern China (aOR: 3.81, 95%CI: 1.38-10.50, *P =* 0.010; aOR: 2.75, 95%CI: 1.18-6.39, *P =* 0.019) (Table 4).

**Table 4:** Task participation among team members of Group B during COVID-19 containment in mainland China

Characteristics	Task participation		Unadjusted		Adjusted	
	No [n (%)]	Yes [n (%)]	OR (95%CI)	***P value***	OR (95%CI)	***P value***
***Pre-examination/triage***						
**Specialty**						
General medicine	0	7 (100)	–	–	–	–
Clinical medicine	15 (30.61)	34 (69.39)	3.26 (1.53-6.94)	0.002*	2.54 (1.05-6.13)	0.038*
Traditional Chinese medicine	9 (27.27)	24 (72.73)	3.83 (1.58-9.33)	0.003*	4.15 (1.5-11.5)	0.006*
Nursing	36 (31.86)	77 (68.14)	3.07 (1.69-5.6)	< 0.001*	3.63 (1.71-7.69)	0.001*
Others	46 (58.97)	32 (41.03)	1	–	1	–
**Region**						
Eastern	34 (68)	16 (32)	1	–	1	–
Central	11 (23.91)	35 (76.09)	6.76 (2.75-16.65)	< 0.001*	3.81 (1.38-10.5)	0.010*
Western	61 (33.15)	123 (66.85)	4.28 (2.2-8.36)	< 0.001*	2.75 (1.18-6.39)	0.019*
**Intro-city location**						
Urban	71 (43.56)	92 (56.44)	1	–	1	–
Urban-rural	31 (35.23)	57 (64.77)	1.42 (0.83-2.43)	0.201	1.2 (0.63-2.27)	0.585
Rural	4 (13.79)	25 (86.21)	4.82 (1.61-14.49)	0.005*	4.01 (1.19-13.47)	0.025*
***At-home quarantine management***						
**Technical title**						
Junior	118 (71.95)	46 (28.05)	1	–	1	–
Intermediate	31 (50.82)	30 (49.18)	2.48 (1.35-4.55)	0.003*	2.52 (1.13-5.61)	0.023*
Associate senior	3 (37.5)	5 (62.5)	4.28 (0.98-18.62)	0.053	3.37 (0.62-18.44)	0.161
Senior	36 (76.6)	11 (23.4)	–	–	–	–
Others	188 (67.14)	92 (32.86)	0.78 (0.37-1.67)	0.528	1.26 (0.55-2.88)	0.588
**Region**						
Eastern	42 (84)	8 (16)	1	–	1	–
Central	34 (73.91)	12 (26.09)	1.85 (0.68-5.05)	0.228	0.94 (0.29-3.08)	0.925
Western	112 (60.87)	72 (39.13)	3.37 (1.5-7.6)	0.003*	2.86 (1.1-7.42)	0.031*
***Screening at travel centers/intervals***						
**Region**						
Eastern	42 (84)	8 (16)	1	–	1	–
Central	28 (60.87)	18 (39.13)	3.37 (1.29-8.82)	0.013*	3.42 (1.18-9.92)	0.023*
Western	147 (79.89)	37 (20.11)	1.32 (0.57-3.05)	0.514	1 (0.37-2.7)	0.992
***Screening at entry centers***						
**Region**						
Eastern	50 (100)	0	–	–	–	–
Central	42 (91.3)	4 (8.7)	5.75 (1.24-26.65)	0.025*	10.37 (1.76-60.92)	0.010*
Western	181 (98.37)	3 (1.63)	1	–	1	–

Unadjusted and adjusted logistic regression analyses were used and variables in univariate analyses including: age, sex, educational levels, specialty, technical titles, years of work experience, economic area locations and intro-city locations of their PHC facilities, and the highest grade of risk levels the area of PHC facilities ever reached

Note: Only listed the results which were statistically significant in adjusted analysis. Table S5 showed all of the results which were statistically significant in un-adjusted analysis

*P < 0.05. CI Confidence interval

Members of Group B who had a middle-level technical title were twice more likely to participate in at-home quarantine management compared to those who had a junior title (aOR: 2.52; 95%CI: 1.13-5.61, *P =* 0.023); those who were from western China were more likely to be involved in at-home quarantine management compared to those who were from eastern China, with the aOR: 2.86, (95%CI: 1.10-7.42, *P =* 0.031) (Table 4).

Those who came from central China were three times more likely to participate in screening at travel centers/intervals compared to those who came from eastern China (aOR: 3.42; 95%CI: 1.18-9.92, *P =* 0.023). Moreover, those who were from central China were less likely to be involved in screening at entry centers compared to those who were from eastern China, with the aOR: 10.37, (95%CI: 1.76-60.92, *P =* 0.010) (Table 4).

## Discussion

Our research surveyed the tasks fulfilled by COVID-19 prevention and control management teams at PHC facilities in mainland China during the COVID-19 containment period. The most important tasks were selected by both Group A and Group B. Although the results in the task participation analysis showed that Group A was more likely to participate in all the tasks except for screening at entry centers, the consensus on the top 7 most important tasks (selected from 19 tasks) were similar between the two groups.

Our survey found that in the pre-outbreak and outbreak period, screening at travel centers/intervals and entry centers were widely recognized as the most important factor among all the respondents. Unlike border closures and travel restrictions [11, 12], screening at travel intervals or entry centers to identify high-risk individuals who had a history of epidemiological exposure or had a fever was necessary personnel in and out possible. Which could minimize the economic cost of strict traffic control and restricted entry-exit [15]. The adjusted analysis found that members of Group A who were from central and western China were more likely to participated in screening at travel centers/intervals than those from eastern China. The adjusted analysis in Group B also showed that PCPs from central China were more likely to be involved in screening than those from eastern China. The main reason for the regional difference in screening participation might be ascribed to the efforts by a large number of social workers and volunteers [16]. As a team member from western China of the pilot interview summarized, perhaps screening at travel intervals/entry centers in low-risk areas can be performed by well-trained non-specialists instead of HCWs after the outbreak period of COVID-19 pandemic.

Besides, quarantine management both at-home and at centralized centers were also selected as the important tasks throughout the whole time of COVID-19 containment by both groups. In other countries, at-home quarantine might be managed by public health workers [13]. However, our study revealed that more than half of COVID-19 prevention and control team members at PHC facilities were engaged in at-home quarantine management affairs here in mainland China. It is true that PCPs in mainland China are providers for both healthcare and public health services [17]. The results also indicated that team members specialized in general medicine were more likely to be in charge of quarantine management compared to nurses and traditional Chinese physicians. General medicine was a relatively new specialty here in China with a history of only 30 years, but the role of general practitioners (GPs) was valued in primary care systems [18]. Some regional differences were found in our survey. Respondents of Group A from eastern China were more likely to work at centralized centers compared to those from central and western China. The analysis of Group B showed that team members from western China were more likely to participate in at-home quarantine management compared to those from eastern China. The regions of eastern, central and western China were divided mainly based on their geographical locations and economic development [19]. As eastern regions had a higher level of economic development and the higher population density in these areas, at-home quarantine would bring a higher risk of cross infection than centralized quarantine [20]. The centralized quarantine strategy was recommended in areas with a higher population density.

According to our survey, it should be noted that pre-examination/triage and fever sentinel surveillance clinic/fever clinic work became increasingly important as the prevention and control of COVID-19 continues. During the transition from outbreak period to regular prevention and control period, confirmed COVID-19 patients from community transmission were declined and the focus of tasks changed to surveillance. Pre-examination/triage and fever sentinel surveillance clinic/fever clinic works within PHC facilities targeted in individuals who visited for any health problems or public care services. Team members here at PHC facilities routinely screened possible or probable COVID-19 cases through travel history asking, epidemiological risk evaluation and simple biochemical, imaging and physical examinations. The adjusted analysis found that team members with a higher educational background were less involved in pre-examination/triage work compared to those with a technical secondary school educational background. Because pre-examination/triage work mainly focused on travel history asking and temperature taking. However, fever sentinel surveillance clinic/fever clinics needs more experienced physicians to diagnose if there were possible or probable COVID-19 cases. Therefore, the results demonstrated that GPs above the median age were more likely to engage in fever sentinel surveillance clinic/fever clinic work. It should be mentioned that fever sentinel surveillance clinic was an innovative action in dealing with communicable diseases within PHC facilities in China.

We also investigated COVID-19 patient treatment participation among COVID-19 prevention and control team members at PHC facilities. The adjusted analysis revealed that respondents of Group A from high-risk areas were more likely to participate in COVID-19 patient treatment. Risk areas in China was defined by the number of confirmed cases during the last 14 days. As the total number of confirmed cases in China was limited, the patient treatment was not the major task of PHC facilities. The results of consensus on the top 7 most important tasks reflected this actual situation. As for transferring of high-risk populations, another important task selected by team members of COVID-19 prevention and control was associated with the risk levels. Members of Group A from medium- or high-risk areas were more likely to be responsible for the transferring processes.

### Limitations

Firstly, the participants were not randomly sampled; stratified purposive sampling was utilized to cover all of the regions of mainland China, the areas at different risk levels and in different intro-city locations. Secondly, this cross-sectional study surveyed the tasks of COVID-19 prevention and control team members involved in the mitigation of COVID-19 retrospectively, which might lead to information bias. The survey was carried out online, and only the questions in the questionnaire were addressed although supplementary items were allowed. In addition, after the final questionnaire was developed, a pilot survey should be conducted. During the survey, a supplementary answer was allowed by the respondents in case of any crucial items were missed in our questionnaire but no new topic was raised by the responses. The results supported the content validity of the questionnaire and overcame this shortcoming to some extent.

## Conclusion

In mainland China, team members of COVID-19 prevention and control at PHC facilities were mainly responsible for screening, quarantine, transferring and monitoring during the COVID-19 pandemic. The significance of the pre-examination/triage and fever sentinel surveillance clinic/fever clinic at PHC facilities increased from the outbreak period to the regular prevention and control period. Team members with lower educational background were competent in pre-examination/triage works but fever sentinel surveillance clinic/fever clinics work should be managed by more experienced general practitioners. The necessity of COVID-19 prevention and control management teams to participate in screening at travel centers/intervals remains to be further discussed.

## Supplementary Information

**Additional file 1.**

## Data Availability

Because the current study was only one section of the research on COVID-19 Prevention and Control in Primary Health Care Facilities in China and the other sections are still under analysis and waiting for future publications. The datasets generated and analysed during the current study are not publicly available but are available from the corresponding author on reasonable request.

## References

[1] Coma E, Mora N, Méndez L (2020). Primary care in the time of COVID-19: monitoring the effect of the pandemic and the lockdown measures on 34 quality of care indicators calculated for 288 primary care practices covering about 6 million people in Catalonia. BMC Fam Pract.

[2] Zeber JE, Khanna N (2021). Primary care responses to the COVID-19 pandemic. Fam Pract.

[3] Loerinc LB, Scheel AM, Evans ST (2021). Discharge characteristics and care transitions of hospitalized patients with COVID-19. Healthc (Amsterdam, Netherlands).

[4] Daumas RP, Silva GAE, Tasca R (2020). The role of primary care in the Brazilian healthcare system: limits and possibilities for fighting COVID-19. Cadernos de saude publica.

[5] Lam LTM, Chua YX, Tan DHY (2020). Roles and challenges of primary care physicians facing a dual outbreak of COVID-19 and dengue in Singapore. Fam Pract.

[6] Franzosa E, Gorbenko K, Brody AA (2021). "at home, with care": Lessons from new York City home-based primary care practices managing COVID-19. J Am Geriatr Soc.

[7] Garattini L, Badinella Martini M, Mannucci PM (2021). Improving primary care in Europe beyond COVID-19: from telemedicine to organizational reforms. Intern Emerg Med.

[8] Blazey-Martin D, Barnhart E, Gillis J (2020). Primary care population management for COVID-19 patients. J Gen Intern Med.

[9] World Health Organization. Operational considerations for case management of COVID-19 in health facility and community: interim guidance. http://www.euro.who.int/en/health-topics/health-emergencies/coronavirus-covid-19/technical-guidance/2020/operational-considerations-for-case-management-of-covid-19-in-health-facility-and-community-interim-guidance,-19-march-2020. 19 March 2020.

[10] Desborough J, Hall Dykgraaf S, de Toca L (2020). Australia's national COVID-19 primary care response. Med J Aust.

[11] Yeoh DK, Foley DA, Minney-Smith CA (2021). Impact of coronavirus disease 2019 public health measures on detections of influenza and respiratory syncytial virus in children during the 2020 Australian winter. Clin Infect Dis.

[12] Wilder-Smith A, Freedman DO. Isolation, quarantine, social distancing and community containment: pivotal role for old-style public health measures in the novel coronavirus (2019-nCoV) outbreak. J Travel Med. 2020;27(2). 10.1093/jtm/taaa020 [published Online First: 2020/02/14]. 10.1093/jtm/taaa020 PMC7107565 32052841

[13] Miralles O, Sanchez-Rodriguez D, Marco E (2021). Unmet needs, health policies, and actions during the COVID-19 pandemic: a report from six European countries. European geriatric medicine.

[14] Hu SL (2020). New normal and new future: summing up and reflection for the future scientific prevention and control COVID-19 to ensure its enduring and steady progress. Fudan Univ J Med Sci.

[15] Rasheed R, Rizwan A, Javed H (2021). Socio-economic and environmental impacts of COVID-19 pandemic in Pakistan-an integrated analysis. Environ Sci Pollut Res Int.

[16] Wang L. Shanghai Normal University. Function and advantage of community social work during prevention and control of COVID-19: an example from Xuhui District, Shanghai. Dissertation database of China national knowledge infrastructure. (in Chinese).

[17] Tan Q (2021). Nature, behavior and development of primary health care institutions. Academics in China.

[18] Zhou N, Qian D, Qiao X (2021). Development of the general practice school (department) in medical colleges & universities: roles and strategies. Chin Genl Pract.

[19] Liqing Li X, Zhou YZ (2021). Degree of coordination between primary care resource allocation and economic development in eastern, central and western China. Chin Gen Pract.

[20] Zhu P, Tan X (2021). Is compulsory home quarantine less effective than centralized quarantine in controlling the COVID-19 outbreak? Evidence from Hong Kong. Sustain Cities Soc.

