# Supplementary material for: Tasks of COVID-19 prevention and control management teams at primary health care facilities in mainland China: a nationwide online cross-sectional survey
Source: BMC Prim Care. 2022 May 6;23:110. doi: 10.1186/s12875-022-01703-0 (PMC9075923; doi:10.1186/s12875-022-01703-0)
Supplement: Supplementary file 1 — Additional file 1. [file 12875_2022_1703_MOESM1_ESM.docx]

**Suplementary materials**

[Results 1](#_Toc96802451)

[Table S1 Descriptive characteristics of team members of COVID-19 prevention and control management at PHC facilities during COVID-19 containment in mainland China 2](#_Toc96802452)

[Table S2 Task participation among team members of COVID-19 prevention and control management at PHC facilities during COVID-19 containment in mainland China 4](#_Toc96802453)

[Table S3 Task participation of team members of COVID-19 prevention and control management at PHC facilities during COVID-19 containment in mainland China 5](#_Toc96802454)

[Table S4 Task participation among team members of Group A during COVID-19 containment in mainland China 6](#_Toc96802455)

[Table S5 Task participation among team members of Group B during COVID-19 containment in mainland China 15](#_Toc96802456)

[Survey on tasks of primary care during COVID-19 pandemic 24](#_Toc96802457)

## Results

**Baseline information**

Logistic regression analysis was conducted for regression and correlation analyses between Group A and Group B (Table S1). The adjusted analysis found that team members with an undergraduate college educational background were less likely to be from Group B compared to those with a technical secondary school educational background (aOR: 0.38; 95%CI: 0.2-0.72, P < 0.001) (Table 2). Team members being specialized in traditional Chinese medicine, nursing and others were more likely to constitute the Group B compared to those being specialized in general medicine (aOR: 7.23, 95%CI: 2.77-18.84, P < 0.001; aOR: 3.34, 95%CI: 1.4-7.97, P = 0.006; aOR: 3.97, 95%CI: 1.64-9.61, P = 0.002) (Table S1).

Team members who had an intermediate technical title and an associate senior title were less likely to come from Group B compared to those had a junior title (aOR: 0.48; 95%CI: 0.33-0.72, P < 0.001; aOR: 0.12; 95%CI: 0.06-0.27, P < 0.001) (Table 2). Team members who came from western China were more likely to constitute Group B compared to those who came from eastern China (aOR: 5.57; 95%CI: 3.52-8.82, P < 0.001) (Table S1).

In addition, team members who were from urban-rural and rural areas were less likely to come from Group B compared to those who were from urban areas (aOR: 0.48, 95%CI: 0.32-0.73, P < 0.001; aOR: 0.25, 95%CI: 0.15-0.43, P < 0.001) (Table S1).

**Task** **participation**

The adjusted analysis found that participation of fever sentinel surveillance works was less likely to be responsible Group B compared to non-participation of fever sentinel surveillance works (aOR: 0.54; 95%CI: 0.37-0.78, P = 0.001); participation of at-home quarantine management was less likely to be in charge by Group B compared to non-participation of at-home quarantine management (aOR: 0.66; 95%CI: 0.48-0.91, P = 0.010) (Table S2).

Non-participation of centralized quarantine management was less responsible by Group B compared to participation of centralized quarantine management (aOR: 0.27; 95%CI: 0.17-0.42, P < 0.001) (Table S2).

Non-participation of screening at entry centers was nearly fivefold the odds ratio to be in charge by Group B compared to participation of screening at entry centers (aOR: 4.9; 95%CI: 1.55-15.53, P = 0.007) (Table S2). Non-participation of transferring high risk populations was less likely to be in charge by Group B compared to participation of transferring (aOR: 0.20; 95%CI: 0.10-0.39, P < 0.001) (Table S2).

| Table S1 Descriptive characteristics of team members of COVID-19 prevention and control management at PHC facilities during COVID-19 containment in mainland China | | | | | | | | | | |
| --- | --- | --- | --- | --- | --- | --- | --- | --- | --- | --- |
| **Characteristics** | **Group A [n(%)]** | **Group B [n(%)]** | | **Unadjusted** | | | **Adjusted** | | |  |
|  |  |  |  | **OR (95%CI)** | **P value** | | **OR (95%CI)** | **P value** | |  |
| **Age** |  |  | |  |  | |  |  | |  |
| At or under the median age | 379(71.78) | 149(28.22) | | 1 | — | |  |  | |  |
| Over the median age | 339(72.13) | 131(27.87) | | 0.98(0.75-1.3) | 0.903 | |  |  | |  |
| **Sex** |  |  | |  |  | |  |  | |  |
| Male | 277(84.19) | 52(15.81) | | 1 | — | | 1 | — | |  |
| Female | 441(65.92) | 228(34.08) | | 2.75(1.97-3.86) | <0.001* | | 1.87(1.21-2.9) | 0.005* | |  |
| **Educational level** |  |  | |  |  | |  |  | |  |
| Technical secondary school | 30(50.85) | 29(49.15) | | 1 | — | | 1 | — | |  |
| College | 182(58.33) | 130(41.67) | | 0.74(0.42-1.29) | 0.288 | | 0.74(0.39-1.39) | 0.347 | |  |
| Undergraduate college | 473(81.13) | 110(18.87) | | 0.24(0.14-0.42) | <0.001* | | 0.38(0.2-0.72) | 0.003* | |  |
| Graduate school | 23(88.46) | 3(11.54) | | 0.13(0.04-0.5) | 0.003* | | 0.26(0.06-1.19) | 0.083 | |  |
| Others | 10(55.56) | 8(44.44) | | 0.83(0.29-2.39) | 0.727 | | 0.69(0.2-2.39) | 0.555 | |  |
| **Specialty** |  |  | |  |  | |  |  | |  |
| General medicine | 138(95.17) | 7(4.83) | | 1 | — | | 1 | — | |  |
| Clinical medicine | 204(80.63) | 49(19.37) | | 4.74(2.08-10.76) | <0.001* | | 1.9(0.79-4.56) | 0.152 | |  |
| Traditional Chinese medicine | 60(64.52) | 33(35.48) | | 10.84(4.54-25.88) | <0.001* | | 7.23(2.77-18.84) | <0.001* | |  |
| Nursing | 192(62.95) | 113(37.05) | | 11.6(5.24-25.67) | <0.001* | | 3.34(1.4-7.97) | 0.006* | |  |
| Others | 124(61.39) | 78(38.61) | | 12.4(5.51-27.89) | <0.001* | | 3.97(1.64-9.61) | 0.002* | |  |
| **Technical title** |  |  | |  |  | |  |  | |  |
| Junior | 246(60) | 164(40) | | 1 | — | | 1 | — | |  |
| Intermediate | 253(80.57) | 61(19.43) | | 0.36(0.26-0.51) | <0.001* | | 0.48(0.33-0.72) | <0.001* | |  |
| Associate senior | 150(94.94) | 8(5.06) | | 0.08(0.04-0.17) | <0.001* | | 0.12(0.06-0.27) | <0.001* | |  |
| Senior | 25(100) | 0 | | — | — | | — | — | |  |
| Others | 44(48.35) | 47(51.65) | | 1.6(1.02-2.53) | 0.043* | | 1.62(0.9-2.9) | 0.105 | |  |
| **Years of work experience** |  |  | |  |  | |  |  | |  |
| Equal or under the median years | 363(71.32) | 146(28.68) | | 1 | — | |  |  | |  |
| Over the median years | 355(72.6) | 134(27.4) | | 0.94(0.71-1.24) | 0.653 | |  |  | |  |
| **Region** |  |  | |  |  | |  |  | |  |
| Eastern | 263(84.03) | 50(15.97) | | 1 | — | | 1 | — | |  |
| Central | 196(80.99) | 46(19.01) | | 1.23(0.79-1.92) | 0.349 | | 2.41(1.4-4.14) | 0.001* | |  |
| Western | 259(58.47) | 184(41.53) | | 3.74(2.62-5.34) | <0.001* | | 5.57(3.52-8.82) | <0.001* | |  |
| **Intro-city location** |  |  | |  |  | |  |  | |  |
| Urban | 343(67.79) | 163(32.21) | | 1 | — | | 1 | — | |  |
| Urban-rural | 187(68) | 88(32) | | 0.99(0.72-1.36) | 0.951 | | 0.48(0.32-0.73) | 0.001* | |  |
| Rural | 188(86.64) | 29(13.36) | | 0.32(0.21-0.5) | <0.001* | | 0.25(0.15-0.43) | <0.001* | |  |
| **Risk** |  |  | |  |  | |  |  | |  |
| Low-risk | 645(70.34) | 272(29.66) | | 1 | — | | 1 | — | |  |
| Medium-risk | 45(90) | 5(10) | | 0.26(0.1-0.67) | 0.005* | | 0.52(0.18-1.5) | 0.228 | |  |
| High-risk | 28(90.32) | 3(9.68) | | 0.25(0.08-0.84) | 0.025* | | 0.57(0.14-2.3) | 0.426 | |  |
| *P<0.05. CI=confidence interval. Unadjusted and adjusted logistic regression analyses were used. | | | | | | | | | |  |
| Table S2 Task participation among team members of COVID-19 prevention and control management at PHC facilities during COVID-19 containment in mainland China | | | | | | | | | | |
| **Tasks** | | | **No. of Group A (%;** N=718) | | | **No. of Group B (%;** N=280) | | | **P value** | |
| **Patient treatment** | | |  | | |  | | |  | |
| Yes | | | 60(8.36) | | | 14(5) | | | 0.069 | |
| No | | | 658(91.64) | | | 266(95) | | |  | |
| **Pre-examination/triage** | | |  | | |  | | |  | |
| Yes | | | 476(66.30) | | | 174(62.14) | | | 0.216 | |
| No | | | 242(33.70) | | | 106(37.86) | | |  | |
| **Fever sentinel surveillance clinic/fever clinic** | | |  | | |  | | |  | |
| Yes | | | 257(35.79) | | | 72(25.71) | | | <0.001*** | |
| No | | | 271(37.74) | | | 167(59.64) | | |  | |
| Not set^a^ | | | 193(26.88) | | | 41(14.64) | | |  | |
| **At-home quarantine management** | | | | | |  | | |  | |
| Yes | | | 390(54.32) | | | 188(67.14) | | | <0.001*** | |
| No | | | 328(45.68) | | | 92(32.86) | | |  | |
| **Centralized quarantine management** | | | | | |  | | |  | |
| Yes | | | 253(35.24) | | | 29(10.36) | | | <0.001*** | |
| No | | | 465(64.76) | | | 251(89.64) | | |  | |
| **Screening at travel centers/intervals** | | | | | |  | | |  | |
| Yes | | | 244(33.98) | | | 63(22.5) | | | <0.001*** | |
| No | | | 474(66.02) | | | 217(77.5) | | |  | |
| **Screening at entry centers** | | |  | | |  | | |  | |
| Yes | | | 16(2.23) | | | 7(2.5) | | | 0.797 | |
| No | | | 702(97.77) | | | 273(97.5) | | |  | |
| **Transferring** | | |  | | |  | | |  | |
| Yes | | | 156(21.73) | | | 11(3.93) | | | <0.001*** | |
| No | | | 562(78.27) | | | 269(96.07) | | |  | |
| ^a^ fever sentinel surveillance clinic/fever clinic was not set. | | | | | | | | | | |
| ***P<0.001 | | | | | | | | | | |

| Table S3 Task participation of team members of COVID-19 prevention and control management at PHC facilities during COVID-19 containment in mainland China | | | | | | |
| --- | --- | --- | --- | --- | --- | --- |
| **Tasks** | **Group A**  **[n (%)]** | **Group B**  **[n (%)]** | **Unadjusted** | | **Adjusted** | |
|  |  |  | **OR (95%CI)** | **P value** | **OR (95%CI)** | **P value** |
| **Patient treatment** |  |  |  |  |  |  |
| Yes | 60(81.08) | 14(18.92) | 0.58(0.32-1.05) | 0.072 | 0.79(0.41-1.54) | 0.491 |
| No | 658(71.21) | 266(28.79) |  |  |  |  |
| **Pre-examination/triage** |  |  |  |  |  |  |
| Yes | 476(73.23) | 174(26.77) | 0.83(0.63-1.11) | 0.216 | 1.29(0.92-1.81) | 0.133 |
| No | 242(69.54) | 106(30.46) |  |  |  |  |
| **Fever sentinel surveillance clinic/fever clinic** |  |  |  |  |  |  |
| Yes | 271(79.01) | 72(20.99) | 0.4(0.29-0.56) | <0.001* | 0.54(0.37-0.78) | 0.001 |
| No | 254(60.33) | 167(39.67) | 1 | — | 1 | — |
| Not set^a^ | 193(82.48) | 41(17.52) | 0.32(0.22-0.48) | <0.001* | 0.36(0.24-0.55) | <0.001* |
| **At-home quarantine management** | |  |  |  |  |  |
| Yes | 390(80.91) | 92(19.09) | 0.41(0.31-0.55) | <0.001* | 0.66(0.48-0.91) | 0.010* |
| No | 328(63.57) | 188(36.43) | 1 | — | 1 | — |
| **Centralized quarantine management** | |  |  |  |  |  |
| No | 465(64.94) | 251(35.06) | 1 | — | 1 | — |
| Yes | 253(89.72) | 29(10.28) | 0.21(0.14-0.32) | <0.001* | 0.27(0.17-0.42) | <0.001* |
| **Screening at travel centers/intervals** | |  |  |  |  |  |
| No | 474(68.6) | 217(31.4) | 1 | — | 1 | — |
| Yes | 244(79.48) | 63(20.52) | 0.56(0.41-0.78) | <0.001* | 0.71(0.49-1.01) | 0.059 |
| **Screening at entry centers** |  |  |  |  |  |  |
| No | 702(72) | 273(28) | 1 | — | 1 | — |
| Yes | 16(69.57) | 7(30.43) | 1.13(0.46-2.76) | 0.797 | 4.9(1.55-15.53) | 0.007* |
| **Transferring** |  |  |  |  |  |  |
| No | 562(67.63) | 269(32.37) | 1 | — | 1 | — |
| Yes | 156(93.41) | 11(6.59) | 0.15(0.08-0.28) | <0.001* | 0.2(0.1-0.39) | <0.001* |
| ^a^ fever sentinel surveillance clinic/fever clinic was not set. | | | | | | |
| *P<0.05. CI=confidence interval. Unadjusted and adjusted logistic regression analyses were used. | | | | | | |

| Table S4 Task participation among team members of Group A during COVID-19 containment in mainland China | | | | | | | | | | | | | |
| --- | --- | --- | --- | --- | --- | --- | --- | --- | --- | --- | --- | --- | --- |
| **Characteristics** | | **Task participation** | | | | **Unadjusted** | | | | **Adjusted** | | | |
|  |  | **No [n (%)]** | | **Yes [n (%)]** | | **OR (95%CI)** | | **P value** | | **OR (95%CI)** | | **P value** | |
| ***Patient treatment*** | |  | |  | |  | |  | |  | |  | |
| **Sex** | |  | |  | |  | |  | |  | |  | |
| Male | | 246(88.81) | | 31(11.19) | | 0.56(0.33-0.95) | | 0.031* | | 0.49(0.25-0.98) | | 0.043* | |
| Female | | 412(93.42) | | 29(6.58) | | 1 | | — | | 1 | | — | |
| **Specialty** | |  | |  | |  | |  | |  | |  | |
| General medicine | | 123(89.13) | | 15(10.87) | | 1 | | — | |  | |  | |
| Clinical medicine | | 190(93.14) | | 14(6.86) | | 0.60(0.28-1.30) | | 0.195 | | 0.65(0.29-1.45) | | 0.294 | |
| Traditional Chinese medicine | | 55(91.67) | | 5(8.33) | | 0.75(0.26-2.15) | | 0.587 | | 0.86(0.29-2.58) | | 0.792 | |
| Nursing | | 176(91.67) | | 16(8.33) | | 0.75(0.36-1.56) | | 0.437 | | 1.28(0.52-3.19) | | 0.592 | |
| Others | | 114(91.94) | | 10(8.06) | | 0.72(0.31-1.67) | | 0.442 | | 1.07(0.43-2.63) | | 0.891 | |
| **Technical title** | |  | |  | |  | |  | |  | |  | |
| Junior | | 223(90.65) | | 23(9.35) | | 1 | | — | | 1 | | — | |
| Intermediate | | 232(91.70) | | 21(8.30) | | 0.88(0.47-1.63) | | 0.680 | | 0.85(0.45-1.62) | | 0.628 | |
| Associate senior | | 137(91.33) | | 13(8.67) | | 0.92(0.45-1.88) | | 0.819 | | 0.83(0.39-1.76) | | 0.635 | |
| Senior | | 23(92) | | 2(8) | | 0.84(0.19-3.81) | | 0.824 | | 0.79(0.16-3.87) | | 0.773 | |
| Others | | 43(97.73) | | 1(2.27) | | 0.23(0.03-1.71) | | 0.150 | | 0.21(0.03-1.74) | | 0.148 | |
| **Region** | |  | |  | |  | |  | |  | |  | |
| Eastern | | 246(93.54) | | 17(6.46) | | 1 | | — | |  | |  | |
| Central | | 170(86.73) | | 26(13.27) | | 2.21(1.16-4.20) | | 0.015 | | 1.78(0.87-3.63) | | 0.112 | |
| Western | | 242(93.44) | | 17(6.56) | | 1.02(0.51-2.04) | | 0.963 | | 1.02(0.51-2.07) | | 0.946 | |
| **Risk** | |  | |  | |  | |  | |  | |  | |
| Low-risk | | 552(85.58) | | 49(7.60) | | 1 | | — | |  | |  | |
| Medium-risk | | 39(86.76) | | 3(6.67) | | 0.87(0.26-2.90) | | 0.819 | | 0.65(0.19-2.28) | | 0.500 | |
| High-risk | | 26(92.86) | | 8(28.57) | | 4.87(2.04-11.61) | | <0.001* | | 3.28(1.21-8.87) | | 0.019* | |
| ***Pre-examination/triage*** | |  | |  | |  | |  | |  | |  | |
| **Age** | |  | |  | |  | |  | |  | |  | |
| At or under the median age | | 140(36.94) | | 239(63.06) | | 1.36(1.00-1.86) | | 0.053 | | 1.12(0.67-1.87) | | 0.675 | |
| Over the median age | | 102(30.09) | | 237(69.91) | | 1 | | — | | 1 | | — | |
| **Educational level** | |  | |  | |  | |  | |  | |  | |
| Technical secondary school | | 4(13.33) | | 26(86.67) | | 1 | | — | |  | |  | |
| College | | 59(32.42) | | 123(67.58) | | 0.32(0.11-0.96) | | 0.042* | | 0.28(0.09-0.86) | | 0.026* | |
| Undergraduate college | | 165(34.88) | | 308(65.12) | | 0.29(0.10-0.84) | | 0.022* | | 0.30(0.10-0.90) | | 0.031* | |
| Graduate school | | 11(47.83) | | 12(52.17) | | 0.17(0.04-0.64) | | 0.009* | | 0.21(0.05-0.82) | | 0.024* | |
| Others | | 3(30) | | 7(70) | | 0.36(0.06-1.99) | | 0.241 | | 0.37(0.06-2.16) | | 0.272 | |
| **Specialty** | |  | |  | |  | |  | |  | |  | |
| General medicine | | 49(35.51) | | 89(64.49) | | 1 | | — | | 1 | | — | |
| Clinical medicine | | 63(30.88) | | 141(69.12) | | 1.23(0.78-1.95) | | 0.372 | | 1.17(0.73-1.88) | | 0.524 | |
| Traditional Chinese medicine | | 22(36.67) | | 38(63.33) | | 0.95(0.51-1.79) | | 0.876 | | 0.98(0.51-1.86) | | 0.943 | |
| Nursing | | 51(26.56) | | 141(73.44) | | 1.52(0.95-2.44) | | 0.082 | | 1.47(0.89-2.45) | | 0.134 | |
| Others | | 57(45.97) | | 67(54.03) | | 0.65(0.39-1.06) | | 0.086 | | 0.63(0.37-1.07) | | 0.085 | |
| **Years of work experience** | |  | |  | |  | |  | |  | |  | |
| Equal or under the median years | | 136(37.47) | | 227(62.63) | | 1.41(1.03-1.92) | | 0.031* | | 1.19(0.72-1.98) | | 0.492 | |
| Over the median years | | 106(29.86) | | 249(70.14) | | 1 | | — | | 1 | | — | |
| **Region** | |  | |  | |  | |  | |  | |  | |
| Eastern | | 100(38.02) | | 163(61.98) | | 1 | | — | |  | |  | |
| Central | | 56(28.57) | | 140(71.43) | | 1.53(1.03-2.28) | | 0.035* | | 1.52(0.94-2.45) | | 0.085 | |
| Western | | 86(33.20) | | 173(66.80) | | 1.23(0.86-1.77) | | 0.251 | | 1.46(0.97-2.21) | | 0.072 | |
| **Intro-city location** | |  | |  | |  | |  | |  | |  | |
| Urban | | 110(32.07) | | 233(67.93) | | 1 | | — | | 1 | |  | |
| Urban-rural | | 71(37.97) | | 116(62.03) | | 0.77(0.53-1.12) | | 0.172 | | 0.62(0.41-0.96) | | 0.032* | |
| Rural | | 61(32.45) | | 127(67.55) | | 0.98(0.67-1.44) | | 0.929 | | 0.85(0.55-1.32) | | 0.472 | |
| **Risk** | |  | |  | |  | |  | |  | |  | |
| Low-risk | | 219(33.95) | | 426(66.05) | | 1 | | — | |  | |  | |
| Medium-risk | | 17(37.78) | | 28(62.22) | | 0.85(0.45-1.58) | | 0.601 | | 0.76(0.39-1.50) | | 0.439 | |
| High-risk | | 6(21.43) | | 22(78.57) | | 1.88(0.75-4.72) | | 0.176 | | 1.59(0.59-4.27) | | 0.357 | |
| **Fever sentinel surveillance clinic/fever clinic^a^** | |  | |  | |  | |  | |  | |  | |
| **Age** | |  | |  | |  | |  | |  | |  | |
| Equal or under the median age | | 163(57.39) | | 121(42.61) | | 2.22(1.56-3.15) | | <0.001* | | 2.18(1.16-4.08) | | 0.015* | |
| Over the median age | | 91(37.76) | | 150(62.24) | | 1 | | — | | 1 | | — | |
| **Sex** | |  | |  | |  | |  | |  | |  | |
| Male | | 85(39.17) | | 132(60.83) | | 0.53(0.37-0.75) | | <0.001* | | 0.7(0.45-1.09) | | 0.115 | |
| Female | | 169(54.87) | | 139(45.13) | | 1 | | — | | 1 | | — | |
| **Specialty** | |  | |  | |  | |  | |  | |  | |
| General medicine | | 32(31.37) | | 70(68.63) | | 1 | | — | | 1 | | — | |
| Clinical medicine | | 61(41.5) | | 86(58.5) | | 0.64(0.38-1.1) | | 0.105 | | 0.71(0.4-1.26) | | 0.244 | |
| Traditional Chinese medicine | | 18(40.91) | | 26(59.09) | | 0.66(0.32-1.37) | | 0.267 | | 0.68(0.32-1.46) | | 0.322 | |
| Nursing | | 85(59.86) | | 57(40.14) | | 0.31(0.18-0.52) | | <0.001* | | 0.44(0.24-0.81) | | 0.009* | |
| Others | | 58(64.44) | | 32(35.56) | | 0.25(0.14-0.46) | | <0.001* | | 0.3(0.16-0.58) | | <0.001* | |
| **Technical title** | |  | |  | |  | |  | |  | |  | |
| Junior | | 101(53.72) | | 87(46.28) | | 1 | | — | | 1 | | — | |
| Intermediate | | 85(47.22) | | 95(52.78) | | 1.3(0.86-1.95) | | 0.213 | | 1.15(0.72-1.84) | | 0.566 | |
| Associate senior | | 46(42.2) | | 63(57.8) | | 1.59(0.99-2.56) | | 0.056 | | 0.98(0.54-1.78) | | 0.936 | |
| Senior | | 3(20) | | 12(80) | | 4.64(1.27-16.99) | | 0.020* | | 3.01(0.75-11.99) | | 0.119 | |
| Others | | 19(57.58) | | 14(42.42) | | 0.86(0.41-1.81) | | 0.682 | | 1.17(0.52-2.67) | | 0.700 | |
| **Years of work experience** | |  | |  | |  | |  | |  | |  | |
| Equal or under the median years | | 151(54.51) | | 126(45.49) | | 1.69(1.19-2.38) | | 0.003* | | 0.83(0.44-1.56) | | 0.563 | |
| Over the median years | | 103(41.53) | | 145(58.47) | | 1 | | — | | 1 | | — | |
| **Region** | |  | |  | |  | |  | |  | |  | |
| Eastern | | 100(51.81) | | 93(48.19) | | 1 | | — | | 1 | | — | |
| Central | | 60(41.96) | | 83(58.04) | | 1.49(0.96-2.3) | | 0.074 | | 1.34(0.75-2.41) | | 0.321 | |
| Western | | 94(49.74) | | 95(50.26) | | 1.09(0.73-1.62) | | 0.685 | | 1.07(0.64-1.77) | | 0.807 | |
| **Intro-city location** | |  | |  | |  | |  | |  | |  | |
| Urban | | 116(51.1) | | 111(48.9) | | 1 | | — | | 1 | | — | |
| Urban-rural | | 72(48.65) | | 76(51.35) | | 1.1(0.73-1.67) | | 0.642 | | 0.99(0.59-1.68) | | 0.976 | |
| Rural | | 66(44) | | 84(56) | | 1.33(0.88-2.01) | | 0.177 | | 1.05(0.6-1.82) | | 0.869 | |
| **Risk** | |  | |  | |  | |  | |  | |  | |
| Low-risk | | 231(48.84) | | 242(51.16) | | 1 | | — | | 1 | | — | |
| Medium-risk | | 19(55.88) | | 15(44.12) | | 0.75(0.37-1.52) | | 0.429 | | 0.56(0.25-1.27) | | 0.163 | |
| High-risk | | 4(22.22) | | 14(77.78) | | 3.34(1.08-10.3) | | 0.036 | | 2.05(0.6-7.07) | | 0.255 | |
| ***At-home quarantine management*** | |  | |  | |  | |  | |  | |  | |
| **Age** | |  | |  | |  | |  | |  | |  | |
| At or under the median age | | 198(52.24) | | 181(47.76) | | 1.76(1.31-2.37) | | <0.001* | | 1.59(0.96-2.64) | | 0.074 | |
| Over the median age | | 130(38.35) | | 209(61.65) | | 1 | | — | | 1 | | — | |
| **Sex** | |  | |  | |  | |  | |  | |  | |
| Male | | 111(40.07) | | 166(59.93) | | 0.69(0.51-0.94) | | 0.017* | | 0.90(0.62-1.30) | | 0.573 | |
| Female | | 217(49.21) | | 224(50.79) | | 1 | | — | | 1 | | — | |
| **Specialty** | |  | |  | |  | |  | |  | |  | |
| General medicine | | 44(31.88) | | 94(68.12) | | 1 | | — | | 1 | | — | |
| Clinical medicine | | 87(42.65) | | 117(57.35) | | 0.63(0.40-0.99) | | 0.045* | | 0.71(0.44-1.14) | | 0.156 | |
| Traditional Chinese medicine | | 31(51.67) | | 29(48.33) | | 0.44(0.24-0.81) | | 0.009* | | 0.46(0.24-0.86) | | 0.015* | |
| Nursing | | 107(55.73) | | 85(44.27) | | 0.37(0.24-0.59) | | <0.001* | | 0.44(0.26-0.73) | | 0.002* | |
| Others | | 59(47.58) | | 65(52.42) | | 0.52(0.31-0.85) | | 0.010* | | 0.67(0.39-1.15) | | 0.147 | |
| **Technical title** | |  | |  | |  | |  | |  | |  | |
| Junior | | 121(49.19) | | 125(50.81) | | 1 | | — | | 1 | | — | |
| Intermediate | | 114(45.06) | | 139(54.94) | | 1.18(0.83-1.68) | | 0.356 | | 1.07(0.72-1.58) | | 0.741 | |
| Associate senior | | 57(38) | | 93(62) | | 1.58(1.04-2.39) | | 0.030* | | 1.11(0.67-1.84) | | 0.699 | |
| Senior | | 10(40) | | 15(60) | | 1.45(0.63-3.36) | | 0.383 | | 0.85(0.33-2.16) | | 0.728 | |
| Others | | 26(59.09) | | 18(40.91) | | 0.67(0.35-1.28) | | 0.228 | | 0.61(0.30-1.22) | | 0.160 | |
| **Years of work experience** | |  | |  | |  | |  | |  | |  | |
| Equal or under the median years | | 183(50.41) | | 180(49.59) | | 1.47(1.10-1.98) | | 0.010* | | 0.95(0.58-1.58) | | 0.858 | |
| Over the median years | | 145(40.85) | | 210(59.15) | | 1 | | — | | 1 | | — | |
| **Intro-city location** | |  | |  | |  | |  | |  | |  | |
| Urban | | 144(41.98) | | 199(58.02) | | 1 | | — | | 1 | | — | |
| Urban-rural | | 96(51.34) | | 91(48.66) | | 0.69(0.48-0.98) | | 0.039* | | 0.66(0.45-0.95) | | 0.026* | |
| Rural | | 88(46.81) | | 100(53.19) | | 0.82(0.57-1.18) | | 0.284 | | 0.70(0.47-1.03) | | 0.073 | |
| **Risk** | |  | |  | |  | |  | |  | |  | |
| Low-risk | | 403(62.48) | | 340(52.71) | | 1 | | — | | 1 | | — | |
| Medium-risk | | 30(66.67) | | 30(66.67) | | 1.79(0.95-3.40 | | 0.073 | | 1.78(0.90-3.51) | | 0.098 | |
| High-risk | | 14(50) | | 20(71.43) | | 2.24(0.97-5.17) | | 0.058 | | 1.96(0.82-4.70) | | 0.130 | |
| ***Centralized quarantine management*** | |  | |  | |  | |  | |  | |  | |
| **Age** | |  | |  | |  | |  | |  | |  | |
| Equal or under the median age | | 255(67.28) | | 124(32.72) | | 1.26(0.93-1.72) | | 0.135 | | 0.94(0.63-1.40) | | 0.764 | |
| Over the median age | | 210(61.95) | | 129(38.05) | | 1 | | — | | 1 | | — | |
| **Sex** | |  | |  | |  | |  | |  | |  | |
| Male | | 166(59.93) | | 111(40.07) | | 0.71(0.52-0.97) | | 0.032* | | 0.80(0.55-1.17) | | 0.250 | |
| Female | | 299(67.80) | | 142(32.20) | | 1 | | — | | 1 | | — | |
| **Educational level** | |  | |  | |  | |  | |  | |  | |
| Technical secondary school | | 21(70) | | 9(30) | | 1 | | — | | 1 | | — | |
| College | | 127(69.78) | | 55(30.22) | | 1.01(0.44-2.35) | | 0.981 | | 1.00(0.41-2.41) | | 0.999 | |
| Undergraduate college | | 299(63.21) | | 174(36.79) | | 1.36(0.61-3.03) | | 0.455 | | 0.90(0.37-2.15) | | 0.804 | |
| Graduate school | | 9(39.13) | | 14(60.87) | | 3.63(1.16-11.41) | | 0.027* | | 1.86(0.54-6.42) | | 0.324 | |
| Others | | 9(90) | | 1(10) | | 0.26(0.03-2.36) | | 0.231 | | 0.18(0.02-1.78) | | 0.143 | |
| **Specialty** | |  | |  | |  | |  | |  | |  | |
| General medicine | | 73(52.9) | | 65(47.1) | | 1 | | — | | 1 | | — | |
| Clinical medicine | | 137(67.16) | | 67(32.84) | | 0.55(0.35-0.86) | | 0.008* | | 0.73(0.46-1.18) | | 0.202 | |
| Traditional Chinese medicine | | 39(65) | | 21(35) | | 0.60(0.32-1.13) | | 0.116 | | 0.62(0.32-1.19) | | 0.152 | |
| Nursing | | 140(72.92) | | 52(27.08) | | 0.42(0.26-0.66) | | <0.001* | | 0.52(0.30-0.89) | | 0.017* | |
| Others | | 76(61.29) | | 48(38.71) | | 0.71(0.43-1.16) | | 0.171 | | 0.90(0.52-1.55) | | 0.703 | |
| **Technical title** | |  | |  | |  | |  | |  | |  | |
| Junior | | 182(73.98) | | 64(26.02) | | 1 | | — | | 1 | | — | |
| Intermediate | | 153(60.47) | | 100(39.53) | | 1.86(1.27-2.72) | | 0.001* | | 1.86(1.20-2.87) | | 0.005* | |
| Associate senior | | 86(57.33) | | 64(42.67) | | 2.12(1.38-3.26) | | 0.001* | | 1.96(1.13-3.38) | | 0.016* | |
| Senior | | 14(56) | | 11(44) | | 2.23(0.97-5.17) | | 0.061 | | 1.85(0.70-4.88) | | 0.214 | |
| Others | | 30(68.18) | | 14(31.82) | | 1.33(0.66-2.66) | | 0.425 | | 1.30(0.60-2.81) | | 0.500 | |
| **Region** | |  | |  | |  | |  | |  | |  | |
| Eastern | | 147(55.89) | | 116(44.11) | | 1 | | — | | 1 | | — | |
| Central | | 136(69.39) | | 60(30.61) | | 0.56(0.38-0.83) | | 0.003* | | 0.61(0.38-0.98) | | 0.042* | |
| Western | | 182(70.27) | | 77(29.73) | | 0.54(0.37-0.77) | | 0.001* | | 0.64(0.42-0.97) | | 0.037* | |
| **Intro-city location** | |  | |  | |  | |  | |  | |  | |
| Urban | | 203(59.18) | | 140(40.82) | | 1 | | — | | 1 | | — | |
| Urban-rural | | 128(68.45) | | 59(31.55) | | 0.67(0.46-0.97) | | 0.036* | | 0.76(0.49-1.18) | | 0.228 | |
| Rural | | 134(71.28) | | 54(28.72) | | 0.58(0.40-0.86) | | 0.006* | | 0.65(0.42-1.02) | | 0.059 | |
| **Risk** | |  | |  | |  | |  | |  | |  | |
| Low-risk | | 420(65.12) | | 225(34.88) | | 1 | | — | | 1 | | — | |
| Medium-risk | | 30(66.67) | | 15(33.33) | | 0.93(0.49-1.77) | | 0.833 | | 1.16(0.57-2.35) | | 0.683 | |
| High-risk | | 15(53.57) | | 13(46.43) | | 1.62(0.76-3.46) | | 0.215 | | 1.93(0.80-4.62) | | 0.141 | |
| ***Screening at travel centers/intervals*** | |  | |  | |  | |  | |  | |  | |
| **Age** | |  | |  | |  | |  | |  | |  | |
| Equal or under the median age | | 265(69.92) | | 114(30.08) | | 1.45(1.06-1.97) | | 0.020* | | 1.28(0.77-2.12) | | 0.339 | |
| Over the median age | | 209(61.65) | | 130(38.35) | | 1 | | — | | 1 | | — | |
| **Sex** | |  | |  | |  | |  | |  | |  | |
| Male | | 165(59.57) | | 112(40.43) | | 0.63(0.46-0.86) | | 0.004* | | 0.7(0.5-0.98) | | 0.037* | |
| Female | | 309(70.07) | | 132(29.93) | | 1 | | — | | 1 | | — | |
| **Years of work experience** | |  | |  | |  | |  | |  | |  | |
| Equal or under the median years | | 251(69.15) | | 112(30.85) | | 1.33(0.97-1.81) | | 0.074 | | 1.11(0.67-1.83) | | 0.683 | |
| Over the median years | | 223(62.82) | | 132(37.18) | | 1 | | — | | 1 | | — | |
| **Region** | |  | |  | |  | |  | |  | |  | |
| Eastern | | 191(72.62) | | 72(27.38) | | 1 | | — | | 1 | | — | |
| Central | | 110(56.12) | | 86(43.88) | | 2.07(1.4-3.07) | | <0.001* | | 1.75(1.14-2.7) | | 0.011* | |
| Western | | 173(66.8) | | 86(33.2) | | 1.32(0.91-1.92) | | 0.148 | | 1.63(1.07-2.48) | | 0.024* | |
| **Intro-city location** | |  | |  | |  | |  | |  | |  | |
| Urban | | 232(67.64) | | 111(32.36) | | 1 | | — | | 1 | | — | |
| Urban-rural | | 139(74.33) | | 48(25.67) | | 0.72(0.48-1.08) | | 0.109 | | 0.57(0.37-0.9) | | 0.016* | |
| Rural | | 103(54.79) | | 85(45.21) | | 1.72(1.2-2.49) | | 0.003* | | 1.3(0.86-1.96) | | 0.211 | |
| ***Screening at entry centers*** | |  | |  | |  | |  | |  | |  | |
| **Specialty** | |  | |  | |  | |  | |  | |  | |
| General medicine | | 136(98.55) | | 2(1.45) | | 1 | | — | | 1 | | — | |
| Clinical medicine | | 200(98.04) | | 4(1.96) | | 1.36(0.25-7.53) | | 0.725 | | 1.81(0.3-10.84) | | 0.517 | |
| Traditional Chinese medicine | | 59(98.33) | | 1(1.67) | | 1.15(0.1-12.96) | | 0.908 | | 1.35(0.12-15.58) | | 0.811 | |
| Nursing | | 184(95.83) | | 8(4.17) | | 2.96(0.62-14.14) | | 0.175 | | 3.36(0.67-16.96) | | 0.142 | |
| Others | | 123(99.19) | | 1(0.81) | | 0.55(0.05-6.17) | | 0.630 | | 0.55(0.04-7.31) | | 0.651 | |
| **Technical title** | |  | |  | |  | |  | |  | |  | |
| Junior | | 243(98.78) | | 3(1.22) | | 1 | | — | | 1 | | — | |
| Intermediate | | 245(96.84) | | 8(3.16) | | 2.64(0.69-10.09) | | 0.154 | | 2.45(0.63-9.57) | | 0.197 | |
| Associate senior | | 147(98) | | 3(2) | | 1.65(0.33-8.3) | | 0.541 | | 1.74(0.33-9.06) | | 0.510 | |
| Senior | | 24(96) | | 1(4) | | 3.37(0.34-33.72) | | 0.300 | | 3.83(0.35-42.27) | | 0.273 | |
| Others | | 43(97.73) | | 1(2.27) | | 1.88(0.19-18.53) | | 0.587 | | 3.35(0.3-37.76) | | 0.329 | |
| **Region** | |  | |  | |  | |  | |  | |  | |
| Eastern | | 255(96.96) | | 8(3.04) | | 1 | | — | | 1 | | — | |
| Central | | 194(98.98) | | 2(1.02) | | 0.33(0.07-1.56) | | 0.162 | | 0.39(0.07-2.04) | | 0.264 | |
| Western | | 253(97.68) | | 6(2.32) | | 0.76(0.26-2.21) | | 0.609 | | 0.76(0.21-2.71) | | 0.668 | |
| **Intro-city location** | |  | |  | |  | |  | |  | |  | |
| Urban | | 334(97.38) | | 9(2.62) | | 1 | | — | | 1 | | — | |
| Urban-rural | | 182(97.33) | | 5(2.67) | | 1.02(0.34-3.09) | | 0.973 | | 1.12(0.3-4.2) | | 0.861 | |
| Rural | | 186(98.94) | | 2(1.06) | | 0.4(0.09-1.87) | | 0.243 | | 0.65(0.12-3.4) | | 0.608 | |
| ***Transferring*** | |  | |  | |  | |  | |  | |  | |
| **Sex** | |  | |  | |  | |  | |  | |  | |
| Male | | 198(71.48) | | 79(28.52) | | 0.53(0.37-0.76) | | 0.001* | | 0.53(0.35-0.82) | | 0.004* | |
| Female | | 364(82.54) | | 77(17.46) | | 1 | | — | | 1 | | — | |
| **Specialty** | |  | |  | |  | |  | |  | |  | |
| General medicine | | 101(73.19) | | 37(26.81) | | 1 | | — | | 1 | | — | |
| Clinical medicine | | 162(79.41) | | 42(20.59) | | 0.71(0.43-1.17) | | 0.181 | | 0.84(0.49-1.44) | | 0.532 | |
| Traditional Chinese medicine | | 43(71.67) | | 17(28.33) | | 1.08(0.55-2.12) | | 0.825 | | 1.25(0.62-2.52) | | 0.532 | |
| Nursing | | 160(83.33) | | 32(16.67) | | 0.55(0.32-0.93) | | 0.027* | | 0.93(0.5-1.72) | | 0.807 | |
| Others | | 96(77.42) | | 28(22.58) | | 0.8(0.45-1.4) | | 0.429 | | 1.01(0.54-1.88) | | 0.985 | |
| **Technical title** | |  | |  | |  | |  | |  | |  | |
| Junior | | 202(82.11) | | 44(17.89) | | 1 | | — | | 1 | | — | |
| Intermediate | | 196(77.47) | | 57(22.53) | | 1.34(0.86-2.07) | | 0.198 | | 1.3(0.82-2.05) | | 0.259 | |
| Associate senior | | 113(75.33) | | 37(24.67) | | 1.5(0.92-2.46) | | 0.106 | | 1.31(0.78-2.2) | | 0.311 | |
| Senior | | 17(68) | | 8(32) | | 2.16(0.88-5.32) | | 0.094 | | 2.23(0.86-5.78) | | 0.098 | |
| Others | | 34(77.27) | | 10(22.73) | | 1.35(0.62-2.94) | | 0.449 | | 1.37(0.59-3.16) | | 0.459 | |
| **Region** | |  | |  | |  | |  | |  | |  | |
| Eastern | | 213(80.99) | | 50(19.01) | | 1 | | — | | 1 | | — | |
| Central | | 153(78.06) | | 43(21.94) | | 1.2(0.76-1.89) | | 0.441 | | 0.85(0.5-1.43) | | 0.533 | |
| Western | | 196(75.68) | | 63(24.32) | | 1.37(0.9-2.08) | | 0.141 | | 1.47(0.95-2.25) | | 0.081 | |
| **Risk** | |  | |  | |  | |  | |  | |  | |
| Low-risk | | 516(80) | | 129(20) | | 1 | | — | | 1 | | — | |
| Medium-risk | | 29(64.44) | | 16(35.56) | | 2.21(1.16-4.19) | | 0.015* | | 2.45(1.22-4.91) | | 0.011* | |
| High-risk | | 17(60.71) | | 11(39.29) | | 2.59(1.18-5.66) | | 0.017* | | 3.08(1.28-7.42) | | 0.012* | |
| *P<0.05. CI=confidence interval. | | | | | | | | | | | | | |
| ^a^ Data of fever sentinel surveillance clinic/fever clinic not set were not involved in. | | | | | | | | | | | | | |
| Unadjusted and adjusted logistic regression analyses were used and variables in univariate analyses including: age, sex, educational levels, specialty, technical titles, years of work experience, economic area locations and intro-city locations of their PHC facilities, and the highest grade of risk levels the area of PHC facilities ever reached. | | | | | | | | | | | | | |
| Table S5 Task participation among team members of Group B during COVID-19 containment in mainland China | | | | | | | | | | | | | |
| **Characteristics** | **Task participation** | | | | **Unadjusted** | | | | **Adjusted** | | | |  |
|  | **No [n(%)]** | | **Yes[n(%)]** | | **OR (95%CI)** | | **P value** | | **OR (95%CI)** | | **P value** | |  |
| ***Patient treatment*** |  | |  | |  | |  | |  | |  | |  |
| **Age** |  | |  | |  | |  | |  | |  | |  |
| At or under the median age | 144(96.64) | | 5(3.36) | | 2.12(0.69-6.51) | | 0.187 | | 1.79(0.56-5.77) | | 0.326 | |  |
| Over the median age | 122(93.13) | | 9(6.87) | | 1 | | — | | 1 | | — | |  |
| **Educational level** |  | |  | |  | |  | |  | |  | |  |
| Technical secondary school | 28(96.55) | | 1(3.45) | | 1 | | — | | 1 | | — | |  |
| College | 126(96.92) | | 4(3.08) | | 0.89(0.1-8.26) | | 0.918 | | 0.88(0.09-8.40) | | 0.913 | |  |
| Undergraduate college | 103(93.64) | | 7(6.36) | | 1.9(0.22-16.12) | | 0.555 | | 1.90(0.22-16.27) | | 0.559 | |  |
| Graduate school | 2(66.67) | | 1(33.33) | | 14(0.62-317.38) | | 0.097 | | 13.42(0.58-309.21) | | 0.105 | |  |
| Others | 7(87.5) | | 1(12.5) | | 4(0.22-72.18) | | 0.348 | | 3.20(0.17-59.21) | | 0.435 | |  |
| **Risk** |  | |  | |  | |  | |  | |  | |  |
| Low-risk | 259(95.22) | | 13(4.78) | | 1 | | — | | 1 | | — | |  |
| Medium-risk | 4(80) | | 1(20) | | 4.98(0.52-47.78) | | 0.164 | | 5.58(0.55-57.00) | | 0.147 | |  |
| High-risk | 3(100) | | 0 | | — | | — | | — | | — | |  |
| ***Pre-examination/triage*** |  | |  | |  | |  | |  | |  | |  |
| **Sex** |  | |  | |  | |  | |  | |  | |  |
| Male | 14(26.92) | | 38(73.08) | | 0.54(0.28-1.06) | | 0.074 | | 0.5(0.22-1.14) | | 0.101 | |  |
| Female | 92(40.35) | | 136(59.65) | | 1 | | — | | 1 | | — | |  |
| **Educational level** |  | |  | |  | |  | |  | |  | |  |
| Technical secondary school | 17(58.62) | | 12(41.38) | | 1 | | — | | 1 | | — | |  |
| College | 42(32.31) | | 88(67.69) | | 2.97(1.3-6.78) | | 0.010* | | 2.4(0.91-6.29) | | 0.076 | |  |
| Undergraduate college | 39(35.45) | | 71(64.55) | | 2.58(1.12-5.95) | | 0.026* | | 2.14(0.77-5.94) | | 0.146 | |  |
| Graduate school | 2(66.67) | | 1(33.33) | | 0.71(0.06-8.73) | | 0.788 | | 0.63(0.04-10.64) | | 0.751 | |  |
| Others | 6(75) | | 2(25) | | 0.47(0.08-2.75) | | 0.404 | | 1.29(0.18-9.2) | | 0.797 | |  |
| **Specialty** |  | |  | |  | |  | |  | |  | |  |
| General medicine | 0 | | 7(100) | | — | | — | | — | | — | |  |
| Clinical medicine | 15(30.61) | | 34(69.39) | | 3.26(1.53-6.94) | | 0.002* | | 2.54(1.05-6.13) | | 0.038* | |  |
| Traditional Chinese medicine | 9(27.27) | | 24(72.73) | | 3.83(1.58-9.33) | | 0.003* | | 4.15(1.5-11.5) | | 0.006* | |  |
| Nursing | 36(31.86) | | 77(68.14) | | 3.07(1.69-5.6) | | <0.001* | | 3.63(1.71-7.69) | | 0.001* | |  |
| Others | 46(58.97) | | 32(41.03) | | 1 | | — | | 1 | | — | |  |
| **Technical title** |  | |  | |  | |  | |  | |  | |  |
| Junior | 64(39.02) | | 100(60.98) | | 1 | | — | | 1 | | — | |  |
| Intermediate | 19(31.15) | | 42(68.85) | | 1.41(0.76-2.65) | | 0.277 | | 1.13(0.51-2.54) | | 0.759 | |  |
| Associate senior | 1(12.5) | | 7(87.5) | | 4.48(0.54-37.27) | | 0.165 | | 2.4(0.21-26.84) | | 0.477 | |  |
| Senior | 0 | | 0 | | — | | — | | — | | — | |  |
| Others | 22(46.81) | | 25(53.19) | | 0.73(0.38-1.4) | | 0.339 | | 2.21(0.9-5.41) | | 0.083 | |  |
| **Years of work experience** |  | |  | |  | |  | |  | |  | |  |
| Equal or under the median years | 61(41.78) | | 85(58.22) | | 1.42(0.87-2.31) | | 0.158 | | 1.1(0.57-2.11) | | 0.783 | |  |
| Over the median years | 45(33.58) | | 89(66.42) | | 1 | | — | | 1 | | — | |  |
| **Region** |  | |  | |  | |  | |  | |  | |  |
| Eastern | 34(68) | | 16(32) | | 1 | | — | | 1 | | — | |  |
| Central | 11(23.91) | | 35(76.09) | | 6.76(2.75-16.65) | | <0.001* | | 3.81(1.38-10.5) | | 0.010* | |  |
| Western | 61(33.15) | | 123(66.85) | | 4.28(2.2-8.36) | | <0.001* | | 2.75(1.18-6.39) | | 0.019* | |  |
| **Intro-city location** |  | |  | |  | |  | |  | |  | |  |
| Urban | 71(43.56) | | 92(56.44) | | 1 | | — | | 1 | | — | |  |
| Urban-rural | 31(35.23) | | 57(64.77) | | 1.42(0.83-2.43) | | 0.201 | | 1.2(0.63-2.27) | | 0.585 | |  |
| Rural | 4(13.79) | | 25(86.21) | | 4.82(1.61-14.49) | | 0.005* | | 4.01(1.19-13.47) | | 0.025* | |  |
| **Fever sentinel surveillance clinic/fever clinic^a^** |  | |  | |  | |  | |  | |  | |  |
| **Educational level** |  | |  | |  | |  | |  | |  | |  |
| Technical secondary school | 18(81.82) | | 4(18.18) | | 1 | | — | | 1 | | — | |  |
| College | 79(69.91) | | 34(30.09) | | 1.94(0.61-6.15) | | 0.262 | | 1.54(0.44-5.41) | | 0.501 | |  |
| Undergraduate college | 62(65.96) | | 32(34.04) | | 2.32(0.72-7.44) | | 0.156 | | 1.48(0.38-5.72) | | 0.567 | |  |
| Graduate school | 2(66.67) | | 1(33.33) | | 2.25(0.16-31.33) | | 0.546 | | 1.65(0.09-30.9) | | 0.738 | |  |
| Others | 6(85.71) | | 1(14.29) | | 0.75(0.07-8.09) | | 0.813 | | 2.15(0.15-31) | | 0.575 | |  |
| **Specialty** |  | |  | |  | |  | |  | |  | |  |
| General medicine | 0 | | 7(100) | | — | | — | | — | | — | |  |
| Clinical medicine | 25(67.57) | | 12(32.43) | | 2.14(0.86-5.34) | | 0.102 | | 1.41(0.5-4) | | 0.518 | |  |
| Traditional Chinese medicine | 20(71.43) | | 8(28.57) | | 1.78(0.65-4.93) | | 0.264 | | 1.39(0.45-4.31) | | 0.569 | |  |
| Nursing | 64(66.67) | | 32(33.33) | | 2.23(1.07-4.66) | | 0.033* | | 1.68(0.73-3.86) | | 0.221 | |  |
| Others | 58(81.69) | | 13(18.31) | | 1 | | — | | 1 | | — | |  |
| **Technical title** |  | |  | |  | |  | |  | |  | |  |
| Junior | 104(73.24) | | 38(26.76) | | 1 | | — | | 1 | | — | |  |
| Intermediate | 27(51.92) | | 25(48.08) | | 2.53(1.31-4.9) | | 0.006* | | 1.94(0.82-4.59) | | 0.129 | |  |
| Associate senior | 3(50) | | 3(50) | | 2.74(0.53-14.15) | | 0.230 | | 0.52(0.04-7.75) | | 0.638 | |  |
| Senior | 33(84.62) | | 6(15.38) | | — | | — | | — | | — | |  |
| Others | 167(69.87) | | 72(30.13) | | 0.5(0.19-1.28) | | 0.148 | | 0.84(0.26-2.72) | | 0.777 | |  |
| **Years of work experience** |  | |  | |  | |  | |  | |  | |  |
| Equal or under the median years | 94(76.42) | | 29(23.58) | | 1.91(1.09-3.35) | | 0.024* | | 1.23(0.58-2.59) | | 0.586 | |  |
| Over the median years | 73(62.93) | | 43(37.07) | | 1 | | — | | 1 | | — | |  |
| **Region** |  | |  | |  | |  | |  | |  | |  |
| Eastern | 37(88.1) | | 5(11.9) | | 1 | | — | | 1 | | — | |  |
| Central | 27(67.5) | | 13(32.5) | | 3.56(1.13-11.19) | | 0.030* | | 2.19(0.53-8.97) | | 0.277 | |  |
| Western | 103(65.61) | | 54(34.39) | | 3.88(1.44-10.44) | | 0.007* | | 2.7(0.76-9.54) | | 0.124 | |  |
| **Intro-city location** |  | |  | |  | |  | |  | |  | |  |
| Urban | 104(76.47) | | 32(23.53) | | 1 | | — | | 1 | | — | |  |
| Urban-rural | 48(63.16) | | 28(36.84) | | 1.9(1.03-3.5) | | 0.040* | | 1.62(0.79-3.33) | | 0.187 | |  |
| Rural | 15(55.56) | | 12(44.44) | | 2.6(1.1-6.12) | | 0.029* | | 2.35(0.85-6.54) | | 0.101 | |  |
| **Risk** |  | |  | |  | |  | |  | |  | |  |
| Low-risk | 162(70.13) | | 69(29.87) | | 1 | | — | | 1 | | — | |  |
| Medium-risk | 2(40) | | 3(60) | | 3.52(0.58-21.55) | | 0.173 | | 3.68(0.51-26.78) | | 0.198 | |  |
| High-risk | 3(100) | | 0 | | — | | — | | — | | — | |  |
| ***At-home quarantine management*** |  | |  | |  | |  | |  | |  | |  |
| **Age** |  | |  | |  | |  | |  | |  | |  |
| At or under the median age | 105(70.47) | | 44(29.53) | | 1.38(0.84-2.28) | | 0.207 | | 2.82(0.42-19.12) | | 0.288 | |  |
| Over the median age | 83(63.36) | | 48(36.64) | | 1 | | — | | 1 | | — | |  |
| **Sex** |  | |  | |  | |  | |  | |  | |  |
| Male | 31(59.62) | | 21(40.38) | | 0.67(0.36-1.24) | | 0.202 | | 0.78(0.36-1.7) | | 0.532 | |  |
| Female | 157(68.86) | | 71(31.14) | | 1 | | — | | 1 | | — | |  |
| **Educational level** |  | |  | |  | |  | |  | |  | |  |
| Technical secondary school | 23(79.31) | | 6(20.69) | | 1 | | — | | 1 | | — | |  |
| College | 84(64.62) | | 46(35.38) | | 2.1(0.8-5.53) | | 0.133 | | 1.29(0.45-3.68) | | 0.635 | |  |
| Undergraduate college | 73(66.36) | | 37(33.64) | | 1.94(0.73-5.19) | | 0.185 | | 0.86(0.28-2.64) | | 0.790 | |  |
| Graduate school | 2(66.67) | | 1(33.33) | | 1.92(0.15-24.87) | | 0.619 | | 0.74(0.04-12.82) | | 0.837 | |  |
| Others | 6(75) | | 2(25) | | 1.28(0.2-8.01) | | 0.793 | | 2.75(0.32-23.63) | | 0.357 | |  |
| **Specialty** |  | |  | |  | |  | |  | |  | |  |
| General medicine | 3(42.86) | | 4(57.14) | | — | | — | | — | | — | |  |
| Clinical medicine | 29(59.18) | | 20(40.82) | | 0.52(0.1-2.57) | | 0.42 | | 0.41(0.07-2.51) | | 0.336 | |  |
| Traditional Chinese medicine | 18(54.55) | | 15(45.45) | | 0.63(0.12-3.24) | | 0.576 | | 0.51(0.08-3.25) | | 0.475 | |  |
| Nursing | 75(66.37) | | 38(33.63) | | 0.38(0.08-1.78) | | 0.22 | | 0.37(0.06-2.19) | | 0.275 | |  |
| Others | 63(80.77) | | 15(19.23) | | 0.18(0.04-0.88) | | 0.035* | | 0.17(0.03-1.09) | | 0.061 | |  |
| **Technical title** |  | |  | |  | |  | |  | |  | |  |
| Junior | 118(71.95) | | 46(28.05) | | 1 | | — | | 1 | | — | |  |
| Intermediate | 31(50.82) | | 30(49.18) | | 2.48(1.35-4.55) | | 0.003* | | 2.52(1.13-5.61) | | 0.023* | |  |
| Associate senior | 3(37.5) | | 5(62.5) | | 4.28(0.98-18.62) | | 0.053 | | 3.37(0.62-18.44) | | 0.161 | |  |
| Senior | 36(76.6) | | 11(23.4) | | — | | — | | — | | — | |  |
| Others | 188(67.14) | | 92(32.86) | | 0.78(0.37-1.67) | | 0.528 | | 1.26(0.55-2.88) | | 0.588 | |  |
| **Years of work experience** |  | |  | |  | |  | |  | |  | |  |
| Equal or under the median years | 107(73.29) | | 39(26.71) | | 1.8(1.08-2.97) | | 0.023* | | 1.26(0.55-2.88) | | 0.588 | |  |
| Over the median years | 81(60.45) | | 53(39.55) | | 1 | | — | | 1 | | — | |  |
| **Region** |  | |  | |  | |  | |  | |  | |  |
| Eastern | 42(84) | | 8(16) | | 1 | | — | | 1 | | — | |  |
| Central | 34(73.91) | | 12(26.09) | | 1.85(0.68-5.05) | | 0.228 | | 0.94(0.29-3.08) | | 0.925 | |  |
| Western | 112(60.87) | | 72(39.13) | | 3.37(1.5-7.6) | | 0.003* | | 2.86(1.1-7.42) | | 0.031* | |  |
| **Risk** |  | |  | |  | |  | |  | |  | |  |
| Low-risk | 186(68.38) | | 86(31.62) | | 1 | | — | | 1 | | — | |  |
| Medium-risk | 2(40) | | 3(60) | | 3.24(0.53-19.77) | | 0.202 | | 2.82(0.42-19.12) | | 0.288 | |  |
| High-risk | 0 | | 3(100) | | — | | — | | — | | — | |  |
| ***Centralized quarantine management*** |  | |  | |  | |  | |  | |  | |  |
| **Sex** |  | |  | |  | |  | |  | |  | |  |
| Male | 49(94.23) | | 3(5.77) | | 2.1(0.61-7.23) | | 0.238 | | 3.42(0.82-14.29) | | 0.091 | |  |
| Female | 202(88.6) | | 26(11.4) | | 1 | | — | | 1 | | — | |  |
| **Educational level** |  | |  | |  | |  | |  | |  | |  |
| Technical secondary school | 28(96.55) | | 1(3.45) | | 1 | | — | |  | |  | |  |
| College | 112(86.15) | | 18(13.85) | | 4.5(0.58-35.16) | | 0.152 | | 2.78(0.33-23.08) | | 0.344 | |  |
| Undergraduate college | 101(91.82) | | 9(8.18) | | 2.5(0.3-20.54) | | 0.395 | | 1.27(0.14-11.88) | | 0.832 | |  |
| Graduate school | 3(100) | | 0 | | — | | — | | — | | — | |  |
| Others | 7(87.5) | | 1(12.5) | | 4(0.22-72.18) | | 0.348 | | 9.3(0.29-302.49) | | 0.210 | |  |
| **Technical title** |  | |  | |  | |  | |  | |  | |  |
| Junior | 150(91.46) | | 14(8.54) | | 1 | | — | | 1 | | — | |  |
| Intermediate | 54(88.52) | | 7(11.48) | | 1.39(0.53-3.62) | | 0.502 | | 1.53(0.55-4.25) | | 0.418 | |  |
| Associate senior | 6(75) | | 2(25) | | 3.57(0.66-19.38) | | 0.14 | | 4.94(0.7-34.89) | | 0.109 | |  |
| Senior | 41(87.23) | | 6(12.77) | | — | | — | | — | | — | |  |
| Others | 251(89.64) | | 29(10.36) | | 1.57(0.57-4.33) | | 0.386 | | 1.34(0.39-4.59) | | 0.638 | |  |
| **Region** |  | |  | |  | |  | |  | |  | |  |
| Eastern | 49(98) | | 1(2) | | 1 | | — | | 1 | | — | |  |
| Central | 39(84.78) | | 7(15.22) | | 8.79(1.04-74.53) | | 0.046* | | 11.67(0.82-165.39) | | 0.069 | |  |
| Western | 163(88.59) | | 21(11.41) | | 6.31(0.83-48.13) | | 0.075 | | 7.99(0.61-104.05) | | 0.113 | |  |
| **Intro-city location** |  | |  | |  | |  | |  | |  | |  |
| Urban | 150(92.02) | | 13(7.98) | | 1 | | — | | 1 | | — | |  |
| Urban-rural | 76(86.36) | | 12(13.64) | | 1.82(0.79-4.19) | | 0.157 | | 1.5(0.59-3.81) | | 0.392 | |  |
| Rural | 25(86.21) | | 4(13.79) | | 1.85(0.56-6.12) | | 0.316 | | 1.15(0.28-4.66) | | 0.845 | |  |
| **Risk** |  | |  | |  | |  | |  | |  | |  |
| Low-risk | 246(90.44) | | 26(9.56) | | 1 | | — | | 1 | | — | |  |
| Medium-risk | 3(60) | | 2(40) | | 6.31(1.01-39.49) | | 0.049* | | 4.58(0.61-34.39) | | 0.139 | |  |
| High-risk | 251(89.64) | | 29(10.36) | | 4.73(0.41-53.96) | | 0.211 | | 5.35(0.29-98.42) | | 0.259 | |  |
| ***Screening at travel centers/intervals*** |  | |  | |  | |  | |  | |  | |  |
| **Educational level** |  | |  | |  | |  | |  | |  | |  |
| Technical secondary school | 21(72.41) | | 8(27.59) | | 1 | | — | | 1 | | — | |  |
| College | 92(70.77) | | 38(29.23) | | 1.08(0.44-2.66) | | 0.86 | | 1.06(0.4-2.8) | | 0.899 | |  |
| Undergraduate college | 94(85.45) | | 16(14.55) | | 0.45(0.17-1.18) | | 0.104 | | 0.4(0.14-1.15) | | 0.090 | |  |
| Graduate school | 3(100) | | 0 | | — | | — | | — | | — | |  |
| Others | 7(87.5) | | 1(12.5) | | 0.38(0.04-3.55) | | 0.392 | |  | |  | |  |
| **Region** |  | |  | |  | |  | |  | |  | |  |
| Eastern | 42(84) | | 8(16) | | 1 | | — | | 1 | | — | |  |
| Central | 28(60.87) | | 18(39.13) | | 3.37(1.29-8.82) | | 0.013* | | 3.42(1.18-9.92) | | 0.023* | |  |
| Western | 147(79.89) | | 37(20.11) | | 1.32(0.57-3.05) | | 0.514 | | 1(0.37-2.7) | | 0.992 | |  |
| **Intro-city location** |  | |  | |  | |  | |  | |  | |  |
| Urban | 131(80.37) | | 32(19.63) | | 1 | | — | | 1 | | — | |  |
| Urban-rural | 69(78.41) | | 19(21.59) | | 1.13(0.6-2.13) | | 0.713 | | 1.35(0.65-2.8) | | 0.417 | |  |
| Rural | 17(58.62) | | 12(41.38) | | 2.89(1.26-6.65) | | 0.013* | | 2.41(0.96-6.03) | | 0.060 | |  |
| ***Screening at entry centers*** |  | |  | |  | |  | |  | |  | |  |
| **Educational level** |  | |  | |  | |  | |  | |  | |  |
| Technical secondary school | 29(100) | | 0 | | — | | — | |  | |  | |  |
| College | 126(96.92) | | 4(3.08) | | 0.22(0.02-2.26) | | 0.204 | |  | |  | |  |
| Undergraduate college | 108(98.18) | | 2(1.82) | | 0.13(0.01-1.61) | | 0.112 | |  | |  | |  |
| Graduate school | 3(100) | | 0 | | — | | — | |  | |  | |  |
| Others | 7(87.5) | | 1(12.5) | | 1 | | — | |  | |  | |  |
| **Region** |  | |  | |  | |  | |  | |  | |  |
| Eastern | 50(100) | | 0 | | — | | — | | — | | — | |  |
| Central | 42(91.3) | | 4(8.7) | | 5.75(1.24-26.65) | | 0.025* | | 10.37(1.76-60.92) | | 0.010* | |  |
| Western | 181(98.37) | | 3(1.63) | | 1 | | — | | 1 | | — | |  |
| ***Transferring*** |  | |  | |  | |  | |  | |  | |  |
| **Age** |  | |  | |  | |  | |  | |  | |  |
| At or under the median age | 141(94.63) | | 8(5.37) | | 0.41(0.11-1.59) | | 0.199 | | 0.43(0.11-1.76) | | 0.241 | |  |
| Over the median age | 128(97.71) | | 3(2.29) | | 1 | | — | | 1 | | — | |  |
| **Specialty** |  | |  | |  | |  | |  | |  | |  |
| General medicine | 7(100) | | 0 | | — | | — | | — | | — | |  |
| Clinical medicine | 45(91.84) | | 4(8.16) | | 6.84(0.74-63.14) | | 0.09 | | 6.5(0.7-60.61) | | 0.101 | |  |
| Traditional Chinese medicine | 33(100) | | 0 | | — | | — | | — | | — | |  |
| Nursing | 107(94.69) | | 6(5.31) | | 4.32(0.51-36.59) | | 0.18 | | 3.84(0.45-32.98) | | 0.220 | |  |
| Others | 269(96.07) | | 11(3.93) | | 1 | | — | | 1 | | — | |  |
| **Risk** |  | |  | |  | |  | |  | |  | |  |
| Low-risk | 262(96.32) | | 10(3.68) | | 1 | | — | | 1 | | — | |  |
| Medium-risk | 4(80) | | 1(20) | | 6.55(0.67-64.07) | | 0.106 | | 5.39(0.51-56.84) | | 0.161 | |  |
| High-risk | 3(100) | | 0 | | — | | — | | — | | — | |  |
| *P<0.05. CI=confidence interval. | | | | | | | | | | | | |  |
| ^a^ Data of fever sentinel surveillance clinic/fever clinic not set were not involved in. | | | | | | | | | | | | |  |
| Unadjusted and adjusted logistic regression analyses were used and variables in univariate analyses including: age, sex, educational levels, specialty, technical titles, years of work experience, economic area locations and intro-city locations of their PHC facilities, and the highest grade of risk levels the area of PHC facilities ever reached. | | | | | | | | | | | | |  |

## Survey on tasks of primary care during COVID-19 pandemic

**Note:**

**A. Please fill the following form according to your true work experience.**

**B. If you have any problem or advice with this research or the questionnaire, please indicate at the end of the questionnaire.**

**Thank you for the cooperation!**

**Part I Basic demographics**

**Age:** years

**Sex:** female□ male□

**Education:**

Technical secondary school□

College□

Undergraduate college□

Graduate school□

Others□

**Profession:**

Clinical medicine□

General medicine□

Traditional Chinese medicine□

Nursing□

Others□

**Technical title:**

Senior□

Associate senior□

Intermediate□

Junior□

Others□

**Years of work experience:**  years

**The highest level in the COVID-19 that the areas of your CHC facility was identified:**

High-risk area□

Medium -risk area□

Low-risk area□

**The area that your CHC facility located in the city:**

Urban area□

Urban-rural area□

Rural area□

**Your CHC facility comes from:**

Province & region

City & district

**Part Ⅱ Selection and rank of the top 7 most important tasks from the tasks listed below for the three different periods of COVID-19 pandemic.**

**Pre-outbreak period** (the period from the initial report of unexplained pneumonia cases to the lockdown in Wuhan, ranging from December 31,2019 to January 23,2020):

① ② ③ ④ ⑤ ⑥ ⑦

**Outbreak period** (the period from the lockdown in Wuhan to when no newly infected patients were identified in Hubei Province, ranging from January 23, 2020 to March 17, 2020):

① ② ③ ④ ⑤ ⑥ ⑦

**Regular prevention and control period** (after March 17, 2020, when no newly infected patients were identified in Hubei Province):

① ② ③ ④ ⑤ ⑥ ⑦

**Options:**

□Screening at travel centers/intervals

□Screening at entry centers

□At-home quarantine management

□Centralized quarantine management

□Transferring

□Pre-examination/triage

□Fever sentinel surveillance clinic/fever clinic

□COVID-19 patient treatment

□Prevention and control of nosocomial infection within PHC facility

□Prevention and control of COVID-19 at residential communities

□Prevention and control of COVID-19 at functional communities (including schools, factories, and enterprises)

□Routine clinical work

□Routine public health services

□Management of discharged COVID-19 patients

□Disinfection

□Psychological interventions

□Nucleic acid testing (NAT) for severe acute respiratory syndrome coronavirus 2 (SARS-CoV-2)

□Sample collection of SARS-CoV-2 NAT

□Resumption of work, production and schooling

□Others (if the task you think important is not listed above, you can provide a supplementary answer)

**Part Ⅲ Did you participated in the tasks mentioned below？**

**COVID-19 patient treatment:**

Yes□ No□

**Pre-examination/triage:**

Yes□ No□

**Fever sentinel surveillance clinic/fever clinic:**

Yes□ No□

**At-home quarantine management:**

Yes□ No□

**Centralized quarantine management:**

Yes□ No□

**Screening at travel centers/intervals:**

Yes□ No□

**Screening at entry centers:**

Yes□ No□

**Transferring:**

Yes□ No□

Do you think there are any tasks that not included in the questionnaire?

Do you think there are any problem or advice during the prevention and control of COVID-19?
